# *Arabidopsis* miR171-Targeted Scarecrow-Like Proteins Bind to GT *cis*-Elements and Mediate Gibberellin-Regulated Chlorophyll Biosynthesis under Light Conditions

**DOI:** 10.1371/journal.pgen.1004519

**Published:** 2014-08-07

**Authors:** Zhaoxue Ma, Xupeng Hu, Wenjuan Cai, Weihua Huang, Xin Zhou, Qian Luo, Hongquan Yang, Jiawei Wang, Jirong Huang

**Affiliations:** 1 National Key Laboratory of Plant Molecular Genetics, Institute of Plant Physiology and Ecology, Shanghai Institutes for Biological Sciences, Chinese Academy of Sciences, Shanghai, China; 2 University of the Chinese Academy of Sciences, Beijing, China; 3 State Key Laboratory of Genetic Engineering, Institute of Plant Biology, Department of Biochemistry, School of Life Sciences, Fudan University, Shanghai, China; 4 School of Life Sciences and Biotechnology, Shanghai Jiaotong University, Shanghai, China; Peking University, China

## Abstract

An extraordinarily precise regulation of chlorophyll biosynthesis is essential for plant growth and development. However, our knowledge on the complex regulatory mechanisms of chlorophyll biosynthesis is very limited. Previous studies have demonstrated that miR171-targeted scarecrow-like proteins (SCL6/22/27) negatively regulate chlorophyll biosynthesis via an unknown mechanism. Here we showed that SCLs inhibit the expression of the key gene encoding protochlorophyllide oxidoreductase (POR) in light-grown plants, but have no significant effect on protochlorophyllide biosynthesis in etiolated seedlings. Histochemical analysis of β-glucuronidase (GUS) activity in transgenic plants expressing *pSCL27::rSCL27-GUS* revealed that SCL27-GUS accumulates at high levels and suppresses chlorophyll biosynthesis at the leaf basal proliferation region during leaf development. Transient gene expression assays showed that the promoter activity of *PORC* is indeed regulated by SCL27. Consistently, chromatin immunoprecipitation and quantitative PCR assays showed that SCL27 binds to the promoter region of *PORC in vivo*. An electrophoretic mobility shift assay revealed that SCL27 is directly interacted with G(A/G)(A/T)AA(A/T)GT *cis*-elements of the *PORC* promoter. Furthermore, genetic analysis showed that gibberellin (GA)-regulated chlorophyll biosynthesis is mediated, at least in part, by SCLs. We demonstrated that SCL27 interacts with DELLA proteins *in vitro* and *in vivo* by yeast-two-hybrid and coimmunoprecipitation analysis and found that their interaction reduces the binding activity of SCL27 to the *PORC* promoter. Additionally, we showed that SCL27 activates *MIR171* gene expression, forming a feedback regulatory loop. Taken together, our data suggest that the miR171-SCL module is critical for mediating GA-DELLA signaling in the coordinate regulation of chlorophyll biosynthesis and leaf growth in light.

## Author Summary

Chlorophyll biosynthesis is essential for plant growth and development. To date, the regulatory mechanisms of chlorophyll biosynthesis have been well understood only in dark conditions. Previous reports showed that miR171-targeted SCL6/22/27 proteins were involved in chlorophyll biosynthesis. However, the molecular mechanism of SCL action remains unclear. In this study, we found that SCLs negatively regulated chlorophyll biosynthesis though suppressing the expression of the key gene *PROTOCHLOROPHYLLIDE OXIDOREDUCTASE* (*POR*). SCL27 is highly expressed at the basal cell proliferation region of young leaves, suggesting an important role of SCLs in inhibiting chloroplast development before cell expansion. In addition, GT-*cis* elements were required for SCL27 directly binding to the *PORC* promoter. Furthermore, we showed that SCLs mediated GA-regulated chlorophyll biosynthesis through direct interaction with DELLA proteins. The interaction between SCLs and DELLAs reduced the DNA binding activity of SCL27. Our uncovered GA-DELLA-SCL module and its DNA binding targets provide new insights into molecular mechanisms by which chlorophyll biosynthesis and cell proliferation are coordinately regulated during leaf development in response to developmental and environmental cues.

## Introduction

Chlorophylls are complexed with their binding proteins and serve two primary functions in photosynthesis: they trap light energy and transfer it to the reaction centers of photosystems [1], [2]. During light absorption and energy transfer, chlorophylls inevitably generate highly reactive singlet oxygen, particularly under strong light, leading to the inhibition of photosynthesis, plant growth and even to cell death [3], [4]. In addition, many chlorophyll precursors present in their free state are strong photosensitizers that produce reactive oxygen species upon light illumination. Therefore, the chlorophyll biosynthetic pathway is strictly regulated in response to developmental and environmental cues.

It has been well documented that chlorophyll biosynthesis is finely regulated at the multiple steps in the pathway and at both transcriptional and post-transcriptional levels [5]. For example, protochlorophyllide (Pchlide) levels of etiolated seedlings are negatively regulated by the phytochrome-interacting factors PIF1 and PIF3-5 [6]–[9], but positively regulated by two transposase-derived transcription factors, FAR1 (far-red impaired response 1) and FHY3 (far-red elongated hypocotyls 3) [10]; the activity of the key enzyme glutamyl-tRNA reductase (HEMA1) is inhibited directly by heme and the FLU protein via a feedback mechanism [2], [11]–[15], while Mg-chelatase is stimulated by the binding of genomes uncoupled 4 (GUN4) to the ChlH subunit (GUN5) of Mg-chelatase, protoporphyrin IX (PPIX) and Mg-PPIX [16]–[19]. It is worth emphasizing that the activity of the key enzyme Pchlide oxidoreductase (POR) is primarily subject to the transcriptional regulation [8], [20]–[21]. The *Arabidopsis* genome contains three differentially regulated *POR* genes. It has been shown that *PORA* is expressed in etiolated seedlings and its mRNA level drops sharply in light; *PORB* is expressed in both etiolated seedlings and light-grown plants; *PORC* expression is activated by light in a fluence rate-dependent manner [22]–[24]. Available evidence revealed that the expression of *PORA* and *PORB* is regulated by the transcription factors ethylene insensitive 3 (EIN3) and its homolog EIN3-like1 (EIL1) via directly binding to the EBS *cis*-elements in the promoter region [21]. Although *PORC* expression was reported to be directly induced by PIF1 [8], it remains unclear how *PORC* is regulated in light where PIF proteins are degraded.

Gibberellic acid (GA) is an important phytohormone that controls many aspects of plant development and growth via the GA-GID-DELLA signaling module in *Arabidopsis* [25]–[30]. With regard to the chlorophyll biosynthetic pathway, DELLA stabilization in the GA-deficient *ga1-3* mutant leads to increased accumulation of Pchlide and PORs in etiolated seedlings, which are substantially more resistant to photo-oxidative damage after transferred from darkness to light [20]. DELLAs promote Pchlide biosynthesis by repressing the transcriptional activity of PIFs in the dark [20], [31], [32]. In contrast, the molecular mechanism underlying the DELLA-regulated *POR* expression is not fully understood. Recently, the miR171-targeted scarecrow-like (SCL) transcription factors SCL6/SCL6-IV, SCL22/SCL6-III and SCL27/SCL6-II (also known as hairy meristems [HAM] and lost meristems [LOM]) have been demonstrated to play an important role in the proliferation of meristematic cells, polar organization and chlorophyll synthesis [33]–[38]. However, it remains unknown how these SCL proteins control chlorophyll synthesis. Here, we provide convincing evidence that DELLA-regulated *POR* expression is, at least in part, mediated by miR171-targeted SCLs in light.

## Results

### miR171-targeted SCLs regulate chlorophyll biosynthesis via the key enzyme POR

**Figure 1:**
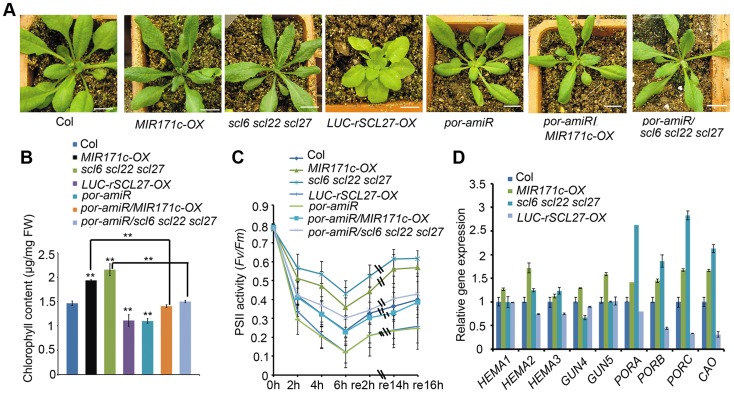
POR is critical for SCL-regulated chlorophyll biosynthesis in light. (A) Phenotypes of WT (Col), *MIR171c-OX*, *scl6 scl22 scl27*, *35S::LUC-rSCL27*, *por-amiR*, *por-amiR/MIR171c-OX* and *por-amiR/scl6 scl22 scl27* plants grown under a 16 h/8 h light/dark cycle. Bars = 1 cm. (B) Chlorophyll content of the genotypes shown in (A). FW, fresh weight. ** indicates *p* values (Student's t-test) <0.01 compared with WT or between the indicated two genotypes. Error bars indicate the s.d. (n = 4). (C) PSII activity (*Fv/Fm*) of the leaves described in (A) treated with excess light (800 µmol m^−2^ s^−1^) for the indicated times and then incubated in the dark. Error bars indicate the s.d. (n = 18). (D) qPCR analysis of *HEMAs*, *GUN4*, *GUN5*, *PORs* and *CAO* transcript levels using total RNA extracted from the leaves of the genotypes shown in (A). The relative expression levels were normalized to that of *ACTIN2*, and the relative expression in WT plants was set as 1. Error bars represent the s.d. (n = 3). Two biological replicates were performed and provided similar results.

As previously reported [38], both *MIR171c* over-expressors (*MIR171c-OX*) and *scl6 scl22 scl27* triple mutants produce dark green leaves (Figure 1A and Figure S1A), which contain approximately 40% more chlorophyll than wild type (WT) leaves (Figure 1B). In contrast, the over-expression of miR171-resistant *LUC-rSCL27* (fused to the luciferase gene) results in leaf yellowing (Figure 1A and Figure S1A) and a significant decrease in chlorophyll content (Figure 1B). These results indicate that miR171-targeted SCLs are negative regulators of chlorophyll biosynthesis.

**Figure 2:**
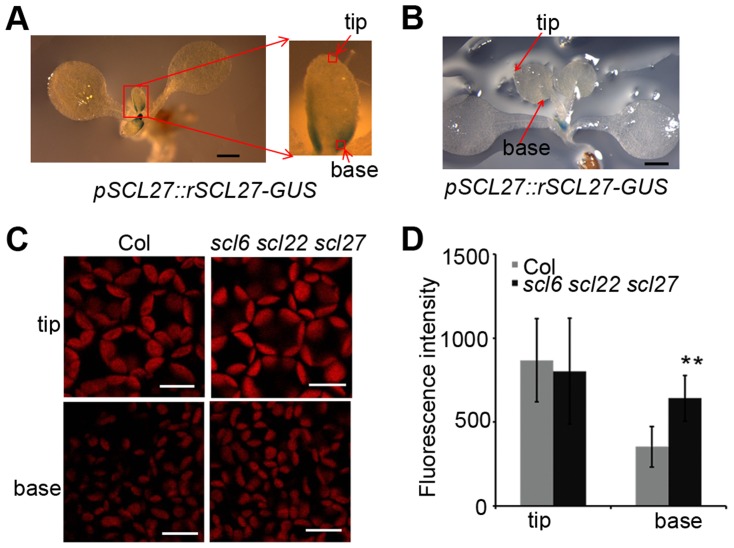
SCL-GUS accumulation and chlorophyll fluorescence intensity at the early stage of leaf growth. (A) The GUS activity of 6-day-old transgenic plants expressing *pSCL27::rSCL27-GUS*. Bar = 1 mm. (B) The GUS activity of 8-day-old transgenic plants expressing *pSCL27::rSCL27-GUS*. Bar = 1 mm. (C) Chlorophyll autofluorescence from the leaf tip and basal cells shown in (A). Bars = 20 µm. (D) Fluorescence intensity in the tip and basal cells of 6-day-old Col and *scl6 scl22 scl27* seedlings. ** represents *p* values (Student's t-test) <0.01 relative to wild-type. Error bars indicate the s.d. (n = 18).

To explore the physiological role of SCL proteins in the regulation of chlorophyll biosynthesis, we constructed transgenic plants expressing *rSCL27* fused to the β-glucuronidase (GUS) gene driven by the *SCL27* native promoter, designated *pSCL27::rSCL27-GUS*. We examined the pattern of GUS expression in the 3- to 11-day-old seedlings. The results of GUS staining clearly showed that the SCL27-GUS fusion protein started to accumulate in the newly developed leaves (Figure S2). In the first pair of leaves, the GUS signal was first observed in the 3-day-old seedlings through the whole leaves, and maintained at a relative stable level at the basal region until to the 7-day-old seedlings, and suddenly disappeared in the 8-day-old seedlings (Figure 2A–2B and Figure S2). Consistent with this observation, the *scl6 scl22 scl27* mutant exhibited more intense chlorophyll fluorescence at the base of leaves than did the WT, whereas chlorophyll fluorescence intensity at the leaf apical region was identical to that in the WT (Figure 2C and 2D), suggesting that SCL proteins play an important role in inhibiting chloroplast development before cell expansion. This result is consistent with a previous report that leaf greening and cell expansion initiate at the leaf tip and proceed in a basipetal direction [39].

We further evaluated the role of SCLs in plant adaptation to high light stress by measuring the ratio of variable fluorescence to maximum fluorescence (*F_v_/F_m_*), which reflects the maximal photochemical efficiency of photosystem II (PSII) photochemistry (PSII activity). Compared to WT plants, PSII activity decreased more slowly in *MIR171c-OX* and *scl6 scl22 scl27* plants but decreased more rapidly in *LUC-rSCL27-OX* plants (Figure 1C) in light stress, indicating that miR171-targeted SCLs are also involved in plant adaptation to excess light. We also investigated the role of SCLs in the growth of etiolated seedlings and chloroplast development. As shown in Figure S3A–S3C, manipulation of *SCL* gene expression slightly but not significantly affected greening ratio, Pchlide content and etioplast ultrastructure of the 5-day-old dark-grown seedlings. However, stacked and stromal thylakoid membranes were thicker in chloroplasts from *MIR171c-OX* and *scl6 scl22 scl27* mature leaves while was thinner in those from *LUC-rSCL27-OX* leaves, compared to WT (Figure S4A–S4B). Consistently, immunoblotting analysis showed that the levels of light-harvesting complex subunits including LHCB1, LHCB2, LHCB5, and LHCA1 were higher in *MIR171c-OX* and *scl6 scl22 scl27* than in WT but lower in *LUC-rSCL27* (Figure S4C). However, changes in SCL expression had no effect on the accumulation of PsaD (PSI subunit) and AtpB (ATP synthase beta subunit) in mature leaves (Figure S4C). Taken together, these results indicate that SCLs are involved in chlorophyll biosynthesis mainly in light but not in the dark.

To elucidate the molecular mechanism underlying SCL-regulated chlorophyll synthesis, we analyzed the transcriptional levels of several key genes in the pathway, including the genes encoding HEMA1, GUN4, GUN5, PORs and chlorophyll *a* oxygenase (CAO). Quantitative PCR (qPCR) and northern blotting assays showed that among the inspected genes the levels of *PORs* and *CAO* transcripts were higher in *MIR171c-OX* and *scl6 scl22 scl27* while were lower in *LUC-rSCL27-OX*, compared to those in the WT (Figure 1D and Figure S1B). The expression levels of *PORs* and *CAO* were correlated well with chlorophyll content in the leaves of *MIR171c-OX*, *scl* triple mutant and *LUC-rSCL27-OX* plants, suggesting that the expression of *PORs* and *CAO* is regulated by SCLs. Immunoblotting analysis using a POR antibody that can recognize all three isoforms of POR showed that *MIR171c-OX* and *scl6 scl22 scl27* plants accumulated higher levels of PORC and PORB proteins than did WT and *LUC-rSCL27-OX* plants (Figure S1C). Thus, the data obtained indicate that the expression of POR, the key enzyme in the chlorophyll biosynthetic pathway, is negatively regulated by SCLs.

To verify the role of POR in SCL-regulated chlorophyll synthesis, we down-regulated the expression of *POR* in WT and *scl6 scl22 scl27* mutant plants using an artificial microRNA that was designed to specifically target the three *POR* genes. Transgenic plants (*por-amiR*) with substantially reduced levels of *POR* expression were identified using qPCR (Figure S1D and S1E). Knocking down *POR* expression in WT, *MIR171c-OX* and *scl* triple mutant plants led to a pale-green phenotype and a lower level of chlorophyll and PSII activity than in the corresponding controls (Figure 1A–1C). Taken together, these data indicate that miR171-targeted SCLs regulate chlorophyll biosynthesis via the key enzyme POR.

### SCL27 binds to the *PORC* promoter

**Figure 3:**
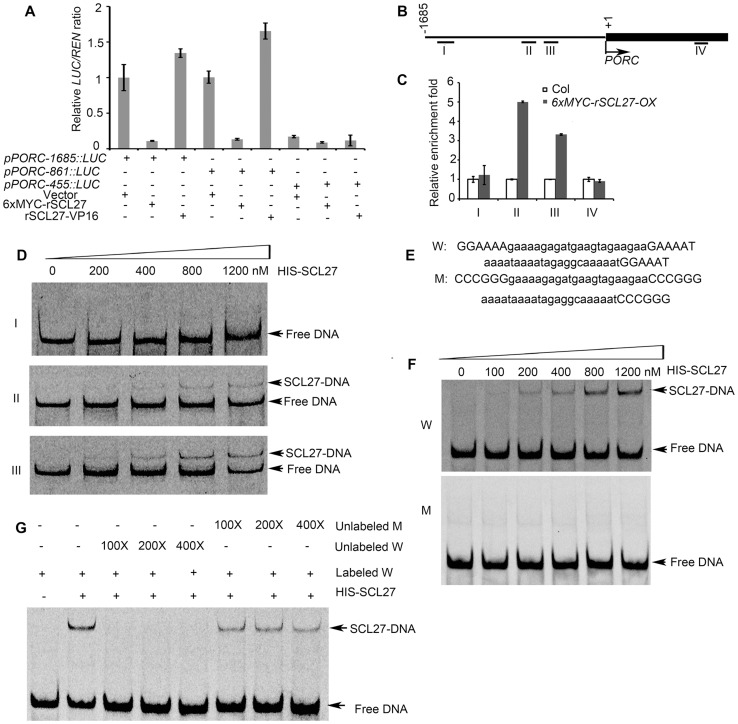
SCL27 binds to the *PORC* promoter. (A) Effect of SCL27 on the activity of three *PORC* promoter regions. The *LUC* reporter gene under the control of these promoter regions was transformed into *N. benthamiana* leaves, with or without *6xMYC-rSCL27* or *rSCL27-VP16*. The relative *LUC* activities were normalized to a *35S::REN* internal control. Error bars indicate the s.d. (n = 4). Three biological replicates provided similar results. (B) Schematic diagram of the *PORC* promoter and the first exon region. Fragments Ι (−1524 bp to −1324 bp), ΙΙ (−778 bp to −598 bp), ΙΙΙ (−572 bp to −372 bp) and IV (1144 bp to 1246 bp) were used for ChIP. (C) ChIP-qPCR analysis of the relative enrichment of the DNA fragments mentioned in (B). The *β-TUBULIN-2* promoter was used as a reference. Error bars indicate the s.d. (n = 3). Two biological replicates were performed and showed similar results. (D) EMSA analysis of SCL27 binding to fragments I, II and III. (E) DNA sequences. W and M contain GT and mutated-GT elements indicated by capital letters, respectively. (F) SCL27 binding to GT elements was analyzed using the indicated levels of purified SCL27 protein mixed with 1 nM of Cy5-fluorescently labeled 62-bp DNA fragments. (G) The specificity of the SCL27-DNA interaction was tested using a competition assay with 0.1, 0.2 and 0.4 µM of unlabeled W or unlabeled M fragments.

The important role of PORs in SCL-regulated chlorophyll biosynthesis prompted us to investigate whether SCLs can directly control the promoter activity of *POR* genes. Because both *PORC* and *MIR171* are regulated by light but not by the circadian clock [22]–[24], [40], we hypothesized that *PORC* was a direct target of SCLs. To test this hypothesis, we co-expressed the *LUC* reporter gene under the control of the *PORC* promoter (a 1685-bp genomic fragment upstream of the start codon) together with 6xMYC-rSCL27 in *Nicotiana benthamiana* leaves using a transient expression system. The expression of *LUC* was much lower in the leaves transformed with *6xMYC*-*rSCL27* than in leaves transformed with the empty vector and *rSCL27-VP16* (a transcriptional activator) (Figure 3A), suggesting that the *PORC* promoter is a direct target of SCL27. To identify the *PORC* promoter region bound by SCL27, three fragments extending from the *PORC* start codon to −1685, −861 and −455 bp upstream were fused to the *LUC* reporter gene. *LUC* expression under the control of either *pPORC-1685* or *pPORC-861* was significantly reduced by 6xMYC-rSCL27 but not by rSCL27-VP16, whereas *LUC* expression driven by *pPORC-455* was low and unaffected by 6xMYC-rSCL27 or rSCL27-VP16 (Figure 3A). Consistently, *LUC* expression under the control of *pPORC-1685* or *pPORC-861* was higher in *MIR171c-OX* and *scl6 scl22 scl27* than in WT (Col), whereas *LUC* expression driven by *pPORC-455* did not significantly differ between WT and *MIR171c-OX* or between WT and *scl6 scl22 scl27* (Figure S5A). These data suggest that the promoter region between −861 bp and −455 bp is required for SCL27 binding to the *PORC* promoter.

We then performed chromatin immuno-precipitation (ChIP) and qPCR assays to further define the SCL27-binding region within the *PORC* promoter (Figure 3B). Our results showed that fragments II (−778 bp to −598 bp) and III (−572 bp to −372 bp) were enriched in immuno-precipitates from the transgenic plants over-expressing *6xMYC-rSCL27* but not in those from WT plants (Figure 3C), whereas fragments I (−1524 bp to −1324 bp) and IV (1144 bp to 1246 bp of the coding sequence, used as a negative control) were not enriched (Figure 3C), indicating that fragments II and III contain SCL27-binding *cis*-elements. We next performed electrophoretic mobility shift assays (EMSAs) to confirm whether SCL27 can directly bind to fragments II and III of the *PORC* promoter. Consistent with the ChIP-qPCR results, shifted bands were observed when purified recombinant SCL27 protein (Figure S5B) was incubated with DNA fragments II or III, and the intensity of the bands gradually increased with increasing concentrations of SCL27 (Figure 3D). However, no shifted band was detected when SCL27 was incubated with fragment I (Figure 3D). Taken together, our *in vivo* and *in vitro* data suggest that SCL27 inhibits *PORC* expression via directly binding to the *PORC* promoter.

GT elements have been reported to be important for light-regulated gene expression, and DNA fragments II and III contain these *cis*-elements [41]. To test whether GT-elements are important for SCL27 binding to the *PORC* promoter, we chose the 62-bp DNA fragment from −500 bp to −438 bp, which contains three G(A/G)(A/T)AA(A/T) GT element repeats [41] (Figure 3E). The EMSA results showed that purified recombinant SCL27 bound to the W fragment but not to the M fragment (Figure 3F). The formation of the SCL27-DNA complex was suppressed by a 100-, 200- or 400-fold excess of unlabeled W fragment, but not by the unlabeled M fragment (Figure 3G). Thus, we conclude that GT elements are required for SCL27 to bind to the *PORC* promoter.

### SCLs mediate DELLA-regulated *POR* expression in light

**Figure 4:**
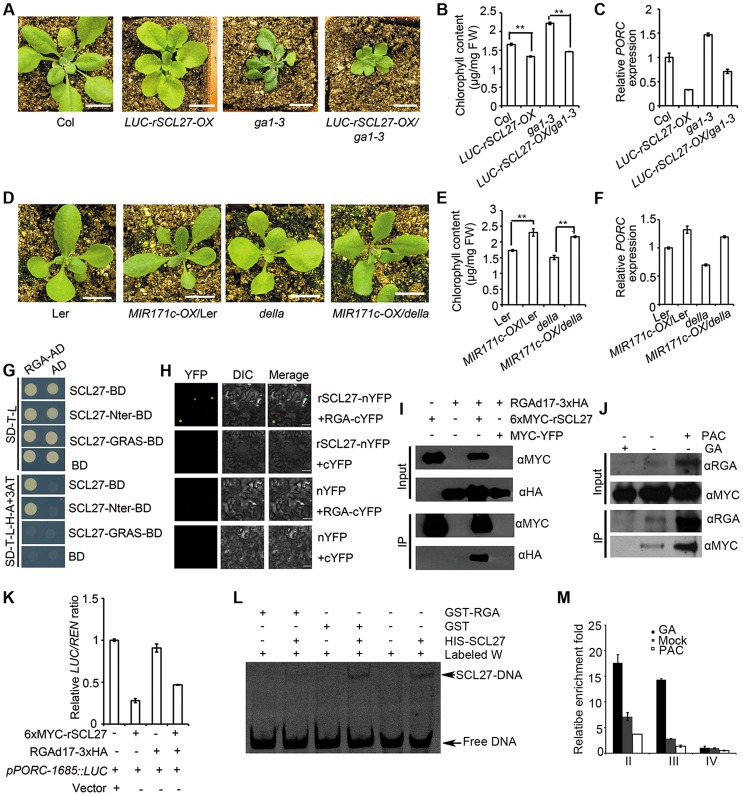
miR171-targeted SCLs mediate DELLA-regulated *POR* expression in light. (A and D) Phenotypes of the indicated plants grown under long-day conditions. Bars = 1 cm. (B, C, E and F) Chlorophyll content (B and E) and relative *PORC* mRNA levels (C and F) of the plants shown in (A and D). ** indicates *p* values (Student's t-test) <0.01 between the indicated two genotypes; error bars indicate the s.d. (n = 4). *PORC* expression levels were normalized to that of *ACTIN2*, and the level of *PORC* expression in Col or L*er* was set as 1. Error bars indicate the s.d. (n = 3). Two biological replicates were performed and showed similar results. FW, fresh weight. (G) Domain mapping of the interaction between SCL27 and RGA in yeast. (H) BiFC analysis of the interaction between SCL27 and RGA. Bars = 50 µm. (I) Co-IP assay of the interaction between SCL27 and RGA using a transient expression assay in *N. benthamiana* leaves. Fusion proteins were detected by immunoblotting with anti-MYC or anti-HA antibodies. (J) Transgenic plants over-expressing *6xMYC-rSCL27* were used for co-IP. *Arabidopsis* proteins were detected by anti-MYC or anti-RGA antibodies. (K) Effect of DELLA binding to SCL27 on *PORC* promoter activity. The *pPORC-1685::LUC* reporter gene was transformed with *6xMYC-rSCL27* and/or *RGAd17-3xHA* in *N. benthamiana*. Relative *LUC* activities were normalized to the *35S::REN* internal control. The *LUC/REN* ratio in the leaves transformed with the vector was set as 1. Error bars indicate the s.d. (n = 4). Three biological replicates showed similar results. (L) The binding of SCL27 to DNA was analyzed using EMSA in the presence of RGA. GST was used as a control. (M) *in vivo* analysis of the binding of SCLs to *PORC* promoter regions in the presence of GA_3_ or PAC. Three-week-old *6xMYC-rSCL27-OX* plants treated with GA or PAC for 2 days were used for ChIP and qPCR experiments. The *β-TUBULIN-2* promoter was used as a reference. Error bars indicate the s.d. (n = 3). Two biological replicates showed similar results.

DELLA proteins up-regulate the expression of *PORs* either in a PIF-dependent or PIF-independent manner [20]. We therefore tested whether miR171-targeted SCLs mediated DELLA-regulated *POR* expression. For this purpose, we generated *LUC-rSCL27-OX*/*ga1-3* or *pSCL27::rSCL27*/*pRGA::RGAd17* (the GA-insensitive form of RGA) plants via sexual crossing. Over-expressing *LUC-rSCL27* in the WT or *ga1-3* genetic background led to pale-green phenotypes and significantly decreased chlorophyll content (Figure 4A, 4B and Figure S6A). Likewise, expressing *pSCR27::rSCL27* in the *pRGA::RGAd17* plants also resulted in a pale-green phenotype and decreased chlorophyll content (Figure S6B and S6C). qPCR analysis showed that over-expressing *LUC-rSCL27* in WT and *ga1-3* plants led to a dramatic decrease in *PORC* expression (Figure 4C). To confirm the epistasis of *SCLs* to *DELLAs* in the regulation of chlorophyll biosynthesis, we over-expressed *MIR171c* in WT (L*er*) and *della* pentuple mutants. Indeed, over-expressing *MIR171c* in these plants resulted in dark green leaves and increased chlorophyll content (Figure 4D and 4E). Consistently, the level of *PORC* expression was higher in *MIR171c* over-expressors than in the corresponding control WT (L*er*) and *della* pentuple plants (Figure 4F). These data indicate that DELLA-promoted chlorophyll biosynthesis and *PORC* expression are dependent on SCLs.

To examine the role of the DELLA-SCL module in chlorophyll biosynthesis in the dark, we measured the greening ratio and Pchlide content of 5-day-old etiolated WT, *MIR171c-OX*, *scl6 scl22 scl27* and *LUC-rSCL27-OX* seedlings in the presence of paclobutrazol (PAC), which increases the levels of DELLA proteins. Our results showed that changes in SCL expression did not affect the greening ratio and Pchlide content in the absence or presence of PAC (Figure S7A and S7B), indicating that SCLs are not involved in DELLA-promoted Pchlide biosynthesis in the dark.

We then tested whether DELLA proteins directly interact with SCLs *in vitro* and *in vivo*. Yeast two-hybrid assays showed that the DELLA protein RGA interacted with SCL27 via the N-terminal domain (Figure 4G). In addition, SCL22 bound to all DELLA proteins in yeast (Figure S8A), indicating that the interaction between SCLs and DELLAs is universal. The *in vivo* interaction between SCLs and DELLAs was examined by bimolecular fluorescence complementation (BiFC) and co-immunoprecipitation (Co-IP) assays using a transient expression system. A strong YFP signal was observed when either rSCL27-nYFP and RGA-cYFP or rSCL27-Nter-nYFP and RGA-cYFP were co-expressed in leaves (Figure 4H and Figure S8B). Co-IP results also showed that RGAd17-3xHA bound to 6xMYC-rSCL27 but not to the control MYC-YFP (Figure 4I). Furthermore, MYC-SCL27 was precipitated by the antibody against RGA in total proteins extracted from transgenic plants over-expressing *6XMYC-rSCL27* treated with PAC but not treated with GA_3_ (Figure 4J). These results demonstrate that RGA interacts directly with SCL27 both *in vitro* and *in vivo*. In addition, qPCR assays showed that the accumulation of *SCL* transcripts was not altered in plants treated with GA_3_ (Figure S9A) or PAC (Figure S9B), or in GA mutants, including *ga1-3*, *gai-2* or *rga gal1 rgl2 rgl3* plants (Figure S9C). Likewise, *RGA* and *GAI* expression was not apparently affected by SCL levels (Figure S9D). Thus, these results exclude the possibility that DELLAs and SCLs are mutually regulated at the transcriptional level.

DELLAs have been shown to regulate various biological processes by preventing transcription factors from binding to DNA [31]–[32], [42]–[46]. The antagonistic role of DELLAs and SCLs in the regulation of chlorophyll biosynthesis raises the possibility that DELLAs might inhibit SCL binding to DNA. To test this hypothesis, we analyzed the promoter activity of *PORC* using a dual-luciferase reporter assay by transforming *RGAd17-3xHA* and/or *6xMYC-rSCL27* into *N. benthamiana* leaves. The results showed that *pPORC-1685::LUC* reporter activity was significantly suppressed by 6xMYC-rSCL27 but was not affected by RGAd17-3xHA. The degree of inhibition of *pPORC-1685::LUC* reporter activity by SCL27 was partially mitigated by the co-expression of RGAd17-3xHA (Figure 4K). Consistent with these results, EMSA analysis showed that the interaction between RGA and SCL27 decreased the binding of SCL27 to DNA (Figure 4L and Figure S10A, S10B). Furthermore, ChIP-qPCR analysis also showed that the enrichment of fragments II and III containing GT elements in the *PORC* promoter (shown in Figure 2B) was higher in MYC antibody pulled-down precipitates from GA-treated *6xMYC-rSCL27-OX* plants but lower in those from PAC-treated *6xMYC-rSCL27-OX* plants than in those from plants given the mock treatment (Figure 4M and Figure S10C). Thus, our data demonstrate that the RGA-SCL27 interaction decreases SCL27 DNA-binding activity.

### SCL27 activates *MIR171* gene expression

**Figure 5:**
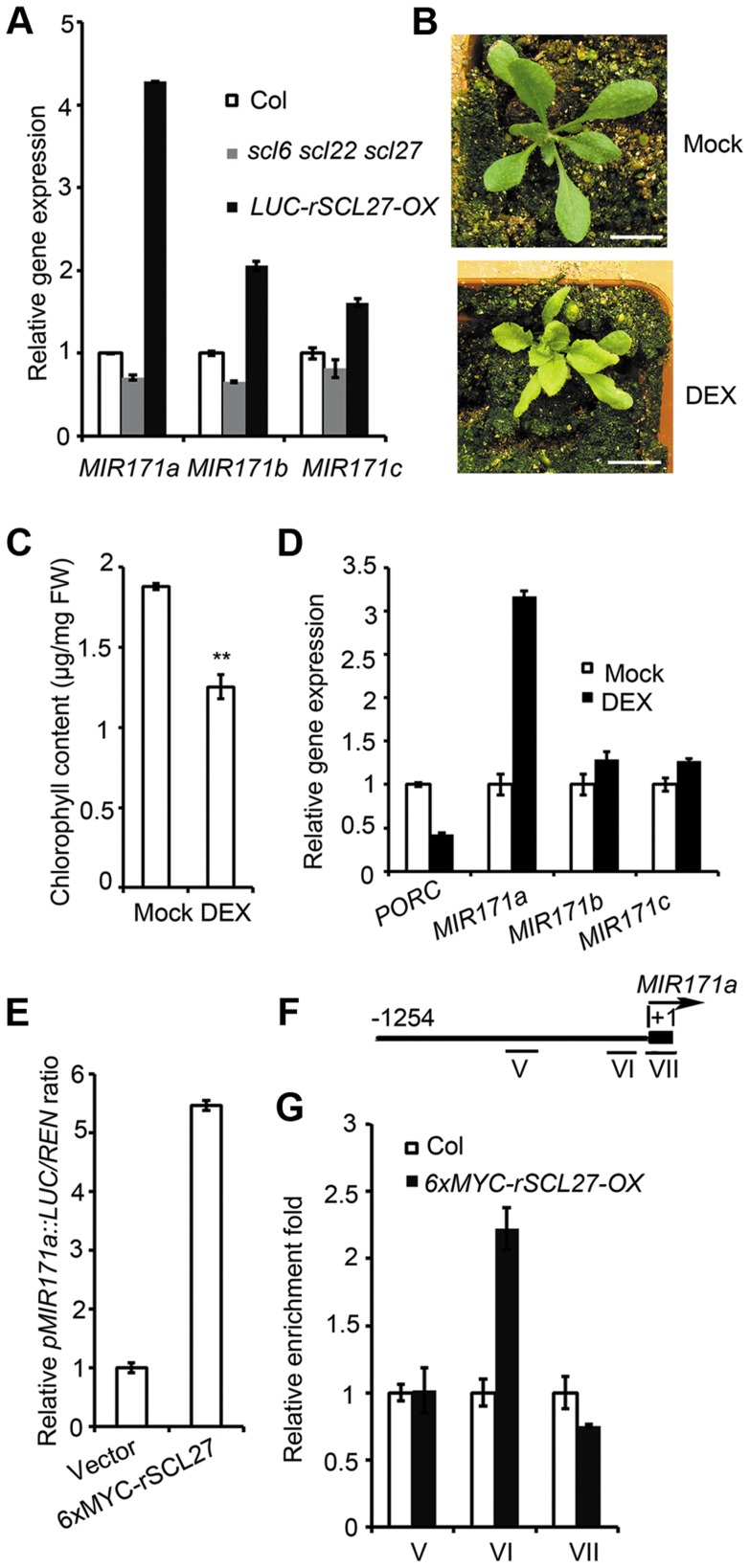
SCL27 activates *MIR171* gene expression in a feedback manner. (A) qPCR analysis of *MIR171a*, *MIR171b* and *MIR171c* expression in Col, *scl6 scl22 scl27* and *LUC-rSCL27-OX* plants. Relative expression levels of *MIR171* genes were normalized to that of *ACTIN2*, and the relative expression in WT plants was set as 1. Error bars represent the s.d. (n = 3). Two biological replicates were performed with similar results. (B) *35S::6xMYC-rSCL27-GR/scl6 scl22 scl27* transgenic plants were treated with DEX (10 µM) or untreated (Mock) for 20 days. Bars = 1 cm. (C) Chlorophyll content of the plants shown in (B). ** indicates *p* value (Student's t-test) <0.01; Error bars indicate the s.d. (n = 4). (D) Relative expression of *PORC*, *MIR171a*, *MIR171b* and *MIR171c* in the plants shown in (B). Expression levels were normalized to that of *ACTIN2*. Expression levels in plants without DEX were set as 1 for each gene. Error bars represent the s.d. (n = 3). Two biological replicates were performed with similar results. (E) Relative activity of the *MIR171a* promoter. *pMIR171a::LUC* was transformed into *N. benthamiana* leaves with or without co-transformation of *6xMYC-rSCL27*. Relative *LUC* activities were normalized to the *35S::REN* internal control. Error bars indicate the s.d. (n = 4). Three biological replicates showed similar results. (F) Schematic diagram of *MIR171a* promoter regions V (−726 bp to −495 bp), VΙ (−260 bp to −71 bp) and VΙΙ (the precursor of *MIR171a*), which were used for ChIP experiments. (G) Relative enrichment of *MIR171a* promoter fragments in the immuno-precipitates. Leaves of 3-week-old Col and *6xMYC-rSCL27-OX* plants were used for ChIP experiments. The enriched DNA fragments were quantified using qPCR. The *β-TUBULIN-2* promoter was used as a reference. Error bars indicate the s.d. (n = 3). Similar results were obtained from three independent immuno-precipitation experiments.

In general, the level of miRNA expression is inversely correlated with the level of target gene expression. However, miR171 accumulation was reported to peak 6 hours earlier than that of *SCL6* [40]. Recent studies have shown that the expression of miRNAs can be controlled by their target genes in a feedback manner [47]. Consistent with this idea, GT elements have been found in the promoters of *MIR171s*. qPCR assays showed that the expression levels of all *MIR171* genes are much higher in *LUC-rSCL27-OX* plants than in WT plants, whereas the expression levels of these genes are lower in *scl6 scl22 scl27* plants than in WT plants (Figure 5A), indicating that SCLs are positive regulators of *MIR171* expression. However, the extent to which SCL27 regulated *MIR171* expression differed among the *MIR171* genes (Figure 5A). For example, SCL27 had the greatest effect on the level of *MIR171a* expression but had lower, similar effects on the expression levels of *MIR171b* and *MIR171c* (Figure 5A).

Additionally, we generated transgenic plants expressing *6xMYC*-*rSCL27* fused to the rat glucocorticoid receptor (GR) under the control of the *35S* regulatory sequence in the *scl* triple mutant background; these plants were designated *35S::6xMYC-rSCL27-GR/scl6 scl22 scl27*. Compared to mock (dimethyl sulfoxide, DMSO)-treated plants, transgenic plants treated with 10 µM dexamethasone (DEX) were pale green and accumulated less chlorophyll (Figure 5B and 5C). qPCR analysis showed that the level of *PORC* mRNA was rapidly decreased in the transgenic plants treated with DEX for 4 hours (Figure 5D). Using this inducible expression system, we found that *MIR171a* transcripts accumulated to levels more than 3-fold higher in DEX-treated plants than in the control, whereas two other *MIR171* genes were only slightly up-regulated by SCL27 (Figure 5D). To confirm the observation that SCL27 activates *MIR171* gene expression, the *LUC* reporter gene driven by the *MIR171a* promoter (*pMIR171a::LUC*) was co-transformed with or without *6xMYC-rSCL27* into *N. benthamiana* leaves. As shown in Figure 5E, *pMIR171a::LUC* activity was significantly increased by 6xMYC-rSCL27. These results indicate that SCLs can up-regulate *MIR171* gene expression. To confirm that SCL27 directly regulates *MIR171* gene expression, ChIP-qPCR was performed using three fragments: V (−726 bp to −495 bp, without GT elements), VI (−260 bp to −71 bp, containing GT elements) and VII (the precursor of *MIR171a*). Indeed, only fragment VI was enriched in MYC antibody pulled-down precipitates obtained from the *6xMYC-rSCL27* over-expressing plants but not in those obtained from the WT plants (Figure 5F and 5G). Taken together, these data clearly indicate that miR171 and its target SCLs form a feedback loop to finely regulate chlorophyll biosynthesis.

## Discussion

miR171 and its target SCL proteins have been reported to play an important role in plant development and growth [33]–[38]. However, little is known about the molecular mechanisms by which the miR171-SCL module functions. In this study, we found compelling evidence showing that SCLs are GT element-binding transcriptional factors that can suppress or promote gene expression in *Arabidopsis*. Given that GT elements are widely distributed in tandem repeats within the promoter regions of many photosynthetic and plastid ribosomal genes [41], it is reasonable to assume that the miR171-SCL module can regulate the expression of other genes in a manner similar to that used for the *PORC* gene.

**Figure 6:**
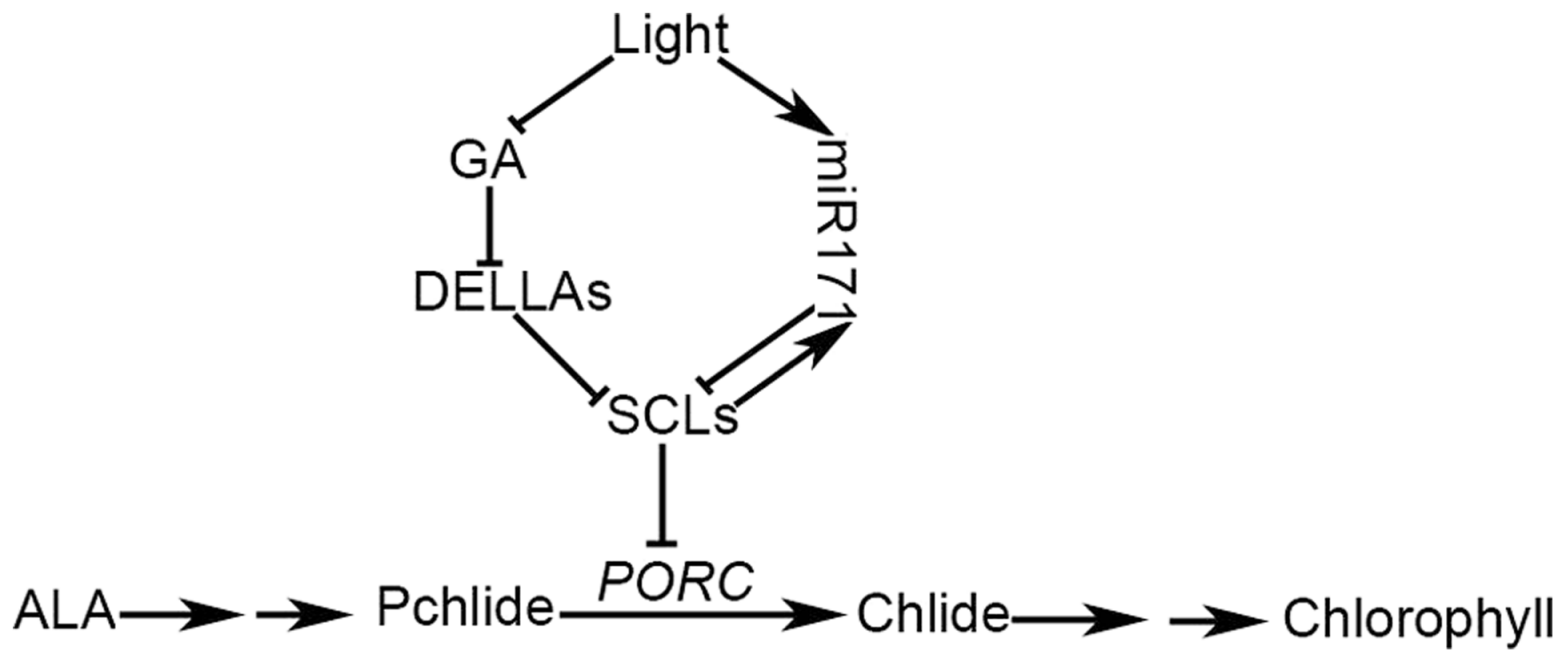
A working model of GA-regulated chlorophyll biosynthesis under the light condition. In light, GA biosynthesis is inhibited, resulting in an increase in DELLAs. Accumulated DELLAs bind to SCLs and subsequently terminate the SCL suppression of *PORC* expression. On the other hand, light promotes miR171 expression, leading to a decrease in *SCL* expression. Thus, the inhibition of SCLs on *PORC* expression is maximally relieved in light. miR171 and its target SCLs form a feedback regulatory loop that maintains the light-dependent diurnal oscillation of miR171 and SCL expression. Arrows indicate verified positive regulation and the chlorophyll biosynthesis pathway; bars indicate verified negative regulation.

In higher plants, light and GA are important signals that antagonistically regulate chloroplast biogenesis, which is a complicated process including chloroplast division and the formation of the photosynthetically active chloroplast [31], [32]. It is well established that PIFs, which are negative regulators of chlorophyll biosynthesis, are critical downstream effectors in light and GA signal transduction pathways [20], [31], [32]. PIFs bind directly to the conserved DNA G-box motif of gene promoters and regulate the chlorophyll biosynthetic pathway by inhibiting Pchlide accumulation and inducing *POR* gene expression in an additive, redundant or specific manner [6]–[9], [20]. This regulatory mechanism involving PIFs is apparently important for the prevention of free Pchlide accumulation and the subsequent greening of etiolated seedlings upon light exposure [20], [31], [32]. Based on the results derived from this study, we suggest that SCLs play an important role in regulating chlorophyll biosynthesis under light conditions (Figure 6), in which PIFs are rapidly degraded. In addition, PIF proteins can be sequestered by DELLAs, the levels of which are elevated in light and decreased in the dark, blocking the ability of PIFs to bind to their target gene promoters [20]. Thus, the SCLs and PIFs control chlorophyll biosynthesis in different yet cooperative manners, and PIFs are replaced by miR171-targeted SCLs to inhibit chlorophyll biosynthesis in light. Since both the levels of DELLA proteins and miR171 expression are elevated in light, the inhibition of SCLs on chlorophyll biosynthesis is coordinately relieved at transcriptional and post-translational levels, while the positive feedback regulation pathway in which SCLs activate miR171 expression might be important for auto-regulating the homeostasis of SCL proteins in light.

In addition to environmental cues, chloroplast development is regulated by developmental signals. Early leaf growth is divided into two sequential cellular processes after primordium initiation: cell proliferation and cell expansion [39]. Usually, chloroplast development is suppressed in the cell proliferation region at the leaf base, then remains relatively stable over a certain period, and finally is abolished abruptly. Once a cell has stopped proliferating, it enters the stage of cell expansion, which is triggered by chloroplast differentiation [39]. Thus, chloroplast differentiation plays an important role in the timing of the transition from cell proliferation to cell expansion. However, blocking chloroplast differentiation and retrograde signaling from chloroplasts to the nuclei using norflurazon cannot completely stop cell expansion, suggesting that other mechanisms are also involved in the phase shift [39]. Our data showed that a negative regulator of chloroplast development, SCL27, is highly expressed at the base of growing leaves, and chloroplast development proceeds more rapidly in a *scl* triple mutant than in WT. These results suggest that miR171-targeted SCLs play an important role in suppressing chloroplast development in dividing cells during early leaf growth. Interestingly, leaf size is apparently altered in *SCL27* over-expressors and the *scl* triple mutant compared to that in WT (Figure 1A). One explanation is that SCLs function as coordinators that simultaneously regulate chloroplast development and cell proliferation; another possibility is that the onset of SCL-regulated chloroplast development leads to a change in the timing of cell proliferation exit. Further investigation is required to elucidate the molecular mechanism by which SCLs coordinately regulate leaf size and chloroplast development.

Chloroplast biogenesis is also coordinated with cell expansion during leaf growth to achieve optimal photosynthesis rates. For example, leaf greening accompanies cell expansion, which initiates at the leaf tip and proceeds in a basipetal direction in *Arabidopsis* [39]. It has been demonstrated that GA plays a critical role in controlling cell expansion and chloroplast biogenesis through DELLA proteins in both dicot and monocot plant species [48]. The number of thylakoid membranes per granum and the chloroplast density per cell are increased in the *ga1-3* mutant, indicating that more chlorophyll is synthesized in the mutant chloroplasts. It is likely that DELLA proteins, which are stabilized in the *ga1-3* mutant, promote chlorophyll biosynthesis by suppressing the inhibitory transcriptional activity of SCLs. Thus, it appears that the DELLA-SCL module functions to balance chloroplast development and cell expansion, which is accompanied by a dramatic increase in photosynthesis.

Furthermore, we observed another complex phenomenon: over-expression of SCLs did not completely rescue the dark-green phenotype of the *ga1-3* mutant, indicating that DELLAs transmit signals that affect chlorophyll biosynthesis by regulating other interacting proteins. A number of DELLA-interacting transcriptional factors have been identified thus far [31]–[32], [42]–[46], including EIN3 and EIL1, which are downstream effectors of ethylene signaling. DELLAs de-repress EIN3 and EIL1 function during apical hook formation in etiolated seedlings [43]. Interestingly, EIN3 and EIL1 were also shown to regulate chlorophyll biosynthesis by repressing the accumulation of Pchlide and by activating the expression of *POR* genes (*PORA* and *PORB*) [21]. It is likely that DELLAs regulate *PORA* and *PORB* expression directly via EIN3 and EIL1. DELLAs might also indirectly regulate chlorophyll biosynthesis through other interacting transcriptional factors, including brassinosteroid-resistant 1 (BZR1) and the jasmonic acid ZIM-domain proteins (JAZs) [42], [49], [50]. Taken together, the findings described here indicate that DELLAs are critical factors integrating various signaling pathways to dynamically regulate chlorophyll biosynthesis.

## Materials and Methods

### Plant materials and growth conditions

*MIR171c-OX*, *scl6 scl22 scl27* triple mutants, *35S::LUC-rSCL27*, *ga1-3*, *gai-2* (SAIL_587_CO_2_), *rga rgl1 rgl2 rgl3* mutants, and *pRGA::RGAd17* are in the *Arabidopsis thaliana* Columbia ecotype (Col) background [27], [38], [44]; the *ga1-3* mutant was backcrossed with the wild type (Col) for six generations; the *por-amiR* and *pSCL27::SCL27-GUS* were transformed in Col background; the *por-amiR/MIR171c-OX*, *LUC-rSCL27-OX/ga1-3*, and *rSCL27/RGAd17* plants were generated by crossing; the *della* pentuple is in the L*er* ecotype [44]. Seeds were germinated and grown on the half Murashige and Skoog (MS) media containing 1% sucrose and 0.7% phytoagar. All plants were grown at 21°C under light (110 µmol. m^−2^. s^−1^) in long days (16-h light/8-h dark).

### Plasmid construction and plant transformation

About 1.7- and 1.2-kb promoter fragments at the upstream of the start codon were amplified from *PORC* and *MIR171a* genes in the Col genome, respectively, with primers listed in Table S1. The amplified fragments were inserted in the *Xho*Ι/*Bam*HI sites of the pGREEN0800LUC vector [51], [52] to produce *pPORC-1685::LUC* and *pMIR171a::LUC* vectors. The *pPORC-861::LUC* and *pPORC-455::LUC* vectors were constructed in a similar way. To make the *POR* amiRNA vector, the amiRNA target sequences for *POR* genes and primers including POR I miR-s, POR II miR-a, POR III miR*s and POR IV miR*a were designed using the WMD3 Web microRNA Designer (http://wmd3.weigelworld.org/cgi-bin/webapp.cgi) and listed in Table S1. The amiRNA precursor was amplified by overlapping PCR from the pRS300 template to produce the fragment containing the *POR* target amiRNA foldback. DNA fragments were gel-purified and cloned into the Gateway cloning vector pENTR-SD/D/TOPO (Invitrogen) according to the manufacturer's instructions. After sequencing confirmation, the cloned DNA fragments were transferred to the *35S* over-expression vector (pGWB2) (Invitrogen) using LR clonase (Invitrogen).

For yeast two-hybrid analysis, *SCL22* cDNA was cloned into the pGBKT7 vector (Clontech). *RGA*, *GAI*, *RGL1*, *RGL2* and *RGL3* cDNAs were cloned into the pGADT7 vector (Clontech). *SCL27* and *RGA* cDNAs were cloned into pDEST22 (Invitrogen); cDNAs encoding SCL27 and its N-terminal (1–267 amino acids) and GRAS domain (268–640 amino acids) were cloned into pDEST32 (Invitrogen). The primers used for these constructs are given in Table S1. For BiFC analysis, *SCL27*, *SCL27 N-terminal*, and *SCL27-GRAS* sequences were cloned into pCAMBIA1300 (nYFP), whereas *RGA* was cloned into pCAMBIA1300 (cYFP).

For *in vitro* protein-DNA binding analysis, *SCL27* and *RGA* was cloned into the pET28b and pGEX6p-3 vectors, respectively. The constructs were transformed into the expression strain BL21 for protein expression. For co-IP analysis, *RGAd17* and miR171-resistent *SCL27* (*rSCL27*) were cloned into the binary vector with 3xHA or 6xMYC. Transgenic plants were generated by the floral dipping method [53] and were screened with 50 mg/mL of kanamycin sulfate or 50 mg/mL of hygromycin.

### Physiological and transmission electron microscopy assays

Seedling greening was analyzed by exposing 5-day-old dark-grown seedlings to white light (16 h-light/8 h-dark) for 2 days. Chlorophyll autofluorescence was analyzed using a confocal laser scanning microscope (Olympus, FV10-ASW). In the PAC-treated etiolated seedlings, 0.01 µM of PAC was used. Greening ratio was determined by counting the percentage of green cotyledons of each genotype. Pchlide was extracted from 5-day-old etiolated seedlings with 1 mL of ice-cold 80% acetone in the dark. The samples were centrifuged at 13000 rpm for 10 min, and fluorescence was excited by the wavelength of 440 nm and scanned from 600 nm to 700 nm using a fluorescence spectrophotometer (Hitachi) at room temperature [54]. The results were presented by relative fluorescence per seedling. Chlorophyll was measured as described previously [55]. The *Fv/Fm* parameter was measured using light-stressed leaf discs after 15-min adaptation to darkness [56]. For electron microscopy observation, cotyledons of 5-day-old etiolated and 25-day-old seedlings were fixed and processed as previously described [57], and examined with an H-7650 transmission electron microscope (Hitachi).

### Yeast two-hybrid assay

Plasmids were transformed into yeast strain AH109 by the lithium chloride–polyethyleneglycol method according to the manufacturer's manual (Clontech). The transformants were selected on SD-Leu-Trp plates. The protein-protein interactions were tested on SD-Trp-Leu -His-Ade plates with or without 3-amino-1, 2, 4-triazole.

### BiFC analysis

The *A. tumefaciens* strain GV3101 transformed with each of the two constructs for BiFC analysis was cultured in the solution containing 10 mM MES, 10 mM MgCl_2,_ and 100 µM acetosyringone to an optical density (OD_600_) of 0.6 to 0.8. Then, two strains were mixed and incubated at the room temperature for at least 2 h. The YFP fluorescence was analyzed using a confocal laser scanning microscope (Olympus, FV10-ASW) 48 to 96 h after *N. benthamiana* leaves were infiltrated with the mixture.

### GUS staining

Plant materials were submerged in 90% acetone for 15 min, and then transferred into 0.5 mg/mL X-Gluc solution (0.1 M sodium phosphate buffer, pH 7.0, 10 mM EDTA, 0.1% Triton X-100, 0.5 mM potassium ferrocyanide, 0.5 mM potassium ferricyanide). Plant materials were vacuumized, kept at 37°C and decolorized in 70% ethanol.

### Co-immunoprecipitation and immunoblot assays

Agrobacteria-infiltrated *N. benthamiana* leaves and transgenic plants over-expressing *6xMYC-rSCL27* were used for Co-IP analyses. The soluble proteins were extracted with the extraction buffer (50 mM Heps [pH 7.5], 150 mM NaCl, 10 mM EDTA [pH 8.0], 0.2% Nonidet P-40, 10% glycerol, 1% PVPP, 2 mM DTT, 1× Complete Protease Inhibitor Cocktail [Sigma]). The beads were washed with the buffer (50 mM Heps [pH 7.5], 200 mM NaCl, 10 mM EDTA [pH 8.0], 0.1% Nonidet P-40, 10% glycerol). Immunoprecipitation was performed with the anti-MYC antibody using *N. benthamiana* leaves. For *Arabidopsis* samples, immunoprecipitation was performed with the anti-RGA antibody. RGAd17-3xHA and 6xMYC-SCL27 fusion proteins were detected by immunoblotting with anti-HA (Sigma) and anti-MYC antibodies (Santa Cruz). To analyze POR, LHCB1, LHCB2, LHCB5, LHCA1, PsaD, and AtpB protein levels *in vivo*, samples (0.1 g) were ground in liquid nitrogen and suspended with 200 µL extraction buffer (125 mM Tris [pH 8.8], 4% SDS, 20% glycerol, 5% β-Me). Total protein was extracted by incubating the samples in boiled water for 5 min, and then centrifuged at 13 000 rpm for 10 min. Proteins were detected with the anti-POR, LHCB1, LHCB2, LHCB5, LHCA1, PsaD, and AtpB antibodies (Agrisera) after total proteins were separated onto a SDS-PAGE gel and transferred to Hybond-ECL Nitrocellulose membrane (Amersham Biosciences).

### Transient transcription dual-luciferase (Dual-LUC) assays

The transient expression assay (Dual-LUC) was carried out as described previously [52]. Agrobacteria-infiltrated *N. benthamiana* leaves were used for *LUC/REN* analyses. Leaf samples were collected for the transient expression assay using commercial Dual-LUC reaction (DLR) reagents, according to the manufacturer's instruction (Promega).

### Quantitative PCR and Northern blot analysis

One µg of total RNAs was used for reverse transcription in a 20 µL reaction system using the RNA PCR (AMV) kit (Promega). Quantitative PCR was performed with SYBR-Green PCR Mastermix (Takara), and amplification was real-time monitored on stepone and steponeplus real-time PCR system (Applied Biosystems). *ACTIN2* was used as an internal control for normalization. The primers are listed in Table S1. Northern blot analysis was carried out as described [58].

### ChIP analysis

ChIP experiments were performed according to published protocols [59]. Briefly, about 3 g tissues of 3-week-old *6xMYC-rSCL27-OX* transgenic plants were harvested. For GA_3_ or PAC treatment, samples were harvested from the plants treated with 10 µM GA_3_ and 0.1 µM PAC for 2 day. After fixation, the materials were resuspended in extraction buffer followed by sonification. One third of the solution was saved as input total DNA without precipitation; another one-third was mixed with the MYC-fused agarose (Sigma); and the remaining one-third was precipitated in parallel with HA-fused agarose as a negative control. The resulting DNA samples were purified using a PCR purification kit (Qiagen). The relative concentrations of the DNA fragments were analyzed by qPCR, using the *β-TUBULIN2* gene promoter as the reference.

### EMSA

The EMSA was performed as reported previously [60]. The primers used were shown in Table S1. The Cy5 fluorescence-labeled DNA (1 nM) was incubated with the indicated amount of the purified His-SCL27 protein in 20 µL of the binding buffer. The concentration of the proteins used for the competitive assay in Figure 4L was 1000 nM. After incubation at 30°C for 20 min, the reaction mixture was electrophoresed at 4°C on a 6% native polyacrylamide gel in 0.5×Tris-borate-EDTA for 2 h (about 200-bp) or 1 h (62-bp) at 100 V. Fluorescence-labeled DNA on the gel was then detected with the Starion FLA-9000 (FujiFilm, Japan).

### Accession numbers

*SCL27* (At2G45160), *SCL22* (At3G60630), *SCL6* (At4G00150), *MIR171A* (At3G51375), *MIR171B* (At1G11735), *MIR171C* (At1G62035), *β-TUBULIN-2* (At5G62690), *HEMA1* (AT1G58290), *GUN4* (AT3G59400), *GUN5* (AT5G13630), *PORA* (AT5G54190), *PORB* (AT4G27440), *PORC* (AT1G03630), *GAI* (AT1G14920), *RGA* (AT2G01570), *RGL1* (AT1G66350), *RGL2* (AT3G03450), and *RGL3* (AT5G17490).

## Supporting Information

Figure S1 Effect of miR171-targeted SCLs on chlorophyll biosynthesis in light. (A) Leaf phenotypes of WT, *MIR171c-OX*, *scl6 scl22 scl27* triple mutant and *LUC-rSCL27-OX* plants grown in long-day conditions. Bar = 0.5 cm. (B) Northern blot analysis of the expression of the indicated genes in (A). Five micrograms of total RNA were loaded on each lane. The levels of rRNAs stained with ethidium bromide are shown as loading controls. (C) Immunodetection of POR levels in (A). (D and E) Relative expression levels of *PORA*, *PORB*, and *PORC* genes in Col and *por-amiR* (D), in *scl6 scl22 scl27* and *por-amiR*/*scl6 scl22 scl27* (E). Expression levels were normalized to that of *ACTIN2*. The expression levels in Col and *scl6 scl22 scl27* were set as 1. Error bars indicate s.d. (n = 3). Two biological replicates were performed with similar results.
(TIF)

Figure S2 GUS staining of transgenic plants *pSCL27::rSCL27-GUS* from 3-day to 11-day seedlings. Bars = 1 mm.
(TIF)

Figure S3 Effect of miR171-targeted SCLs on chlorophyll biosynthesis in the dark. (A) Greening ratio of 5-day-old etiolated seedlings transferred to white light for 2 days. Three biological repeats were performed. Error bars indicate s.d. (n = 30). (B) Pchlide levels of 5-day-old Col, *MIR171c-OX*, *scl6 scl22 scl27*, *LUC-rSCL27-OX* etiolated seedlings. Error bars indicate s.d. (n = 30). Three biological repeats were performed. (C) Ultrastructure of plastids in 5-day-old etiolated seedlings. Bars = 1 µm.
(TIF)

Figure S4 Effect of miR171-targeted SCLs on chloroplast development. (A) Ultrastructure of chloroplasts in mature leaves from 25-day-old plants. Bars = 1 µm. (B) Statistic analysis of stacked and stromal thylakoid membranes. Error bars indicate s.e. (n>110). (C) Immunoblot analysis of PsaD, LHCB1, LHCB2, LHCB5, LHCA1, and AtpB expression in 25-day-old plants.
(PDF)

Figure S5 SCL27 binds to the *PORC* promoter in *Arabidopsis*. (A) The *LUC* reporter gene driven by *pPORC-1685, pPORC-861* or *pPORC-455* was transformed into Col, *MIR171c-OX*, and *scl6 scl22 scl27* plants. The relative *LUC* activities were normalized to the *35S::REN* internal control. Error bars indicate the s.d. (n = 4). Three biological replicates showed similar results. (B) The purified His-SCL27 protein used for EMSA in Figure 3D, 3F, 3G and 4L.
(TIF)

Figure S6 Genetic analysis of *SCL27* and *RGA*. (A) Chlorophyll content of the genotypes shown in Figure 4A based on the total protein (TP). **represent *p* values (Student's t-test) <0.01 relative to wild-type and *ga1-3*, respectively. Error bars indicate s.d. (n = 4). (B) Phenotypes of Col, *pSCL27::rSCL27*, *pRGA::RGAd17*, *pSCL27::rSCL27*/*pRGA::RGAd17* plants grown in long-day conditions for 25 days. Bars = 1 cm. (C) Chlorophyll content of the genotypes shown in (B) based on the fresh weight (FW). ** represent *p* values (Student's t-test) <0.01 relative to wild-type and *pRGA::RGAd17*, respectively. Error bars indicate s.d. (n = 4).
(TIF)

Figure S7 Effect of SCL in DELLA-regulated chlorophyll biosynthesis in the dark. (A) Greening ratio of 5-day-old Col, *MIR171c-OX*, *scl6 scl22 scl27*, *LUC-rSCL27-OX* etiolated seedlings that were grown on the media with PAC or without PAC (methanol, Mock) and transferred to white light for 2 days. Three biological repeats were performed. Error bars indicate s.d. (n = 30). (B) Pchlide levels of 5-day-old Col, *MIR171c-OX*, *scl6 scl22 scl27*, *LUC-rSCL27-OX* etiolated seedlings grown in the media with PAC or without PAC (Mock). Error bars indicate s.d. (n = 3). Three biological repeats were performed.
(TIF)

Figure S8 SCLs interact with DELLAs. (A) SCL22 interacts with DELLAs in yeast. (B) BiFC analysis of the interaction between the N-terminal of SCL27 (SCL27-Nter) and RGA. The following pairs of constructs, *SCL27-Nter-nYFP* and *RGA-cYFP*, *SCL27-Nter-nYFP* and *cYFP*, *SCL27-GRAS-nYFP* and *RGA-cYFP*, and *nYFP* and *cYFP*, were co-transformed into *N. benthamiana* leaves. Bars = 50 µm.
(TIF)

Figure S9 Expression of *SCLs* and *DELLAs* is not affected mutually at the transcriptional level. (A and B) qPCR analysis of *MIR171c*, *SCL27*, *SCL22*, and *SCL6* expression in seedlings treated with GA_3_ or Mock (ethanol) (A), and PAC or Mock (methanol) (B). (C) qPCR analysis of *MIR171c*, *SCL27*, *SCL22*, and *SCL6* expression in GA mutants including *ga1-3*, *gai-2* and *rga rgl1 rgl2 rgl3*. (D) qPCR analysis of *RGA* and *GAI* expression in Col, *MIR171c-OX*, *scl6 scl22 scl27*, *LUC-rSCL27-OX* seedlings. Expression was normalized to that of *ACTIN2* and in WT treated with mock or in WT was set as 1 for each gene. Two biological replicates were performed with similar results. Error bars represent s.d. (n = 3).
(TIF)

Figure S10 RGA reduces the binding activity of SCL27 to the *PORC* promoter. (A and B) The purified GST and GST-RGA proteins used for EMSA in Figure 4L. (C) Relative enrichment of the DNA fragments in the immuno-precipitate related to Figure 4M. Leaves of three-week-old Col plants treated with GA, PAC and Mock (without GA and PAC) were used for ChIP experiments. The obtained DNA fragments were quantified via qPCR. The *β-TUBULIN-2* promoter was used as a reference. Error bars indicate the s.d. (n = 3). Two biological replicates were performed with similar results.
(TIF)

Table S1 A list of primers used in this study.
(DOCX)
