# Supplementary figures and images for: Arabidopsis miR171-Targeted Scarecrow-Like Proteins Bind to GT cis-Elements and Mediate Gibberellin-Regulated Chlorophyll Biosynthesis under Light Conditions
Source: PLoS Genet. 2014 Aug 7;10(8):e1004519. doi: 10.1371/journal.pgen.1004519 (PMC4125095; doi:10.1371/journal.pgen.1004519)

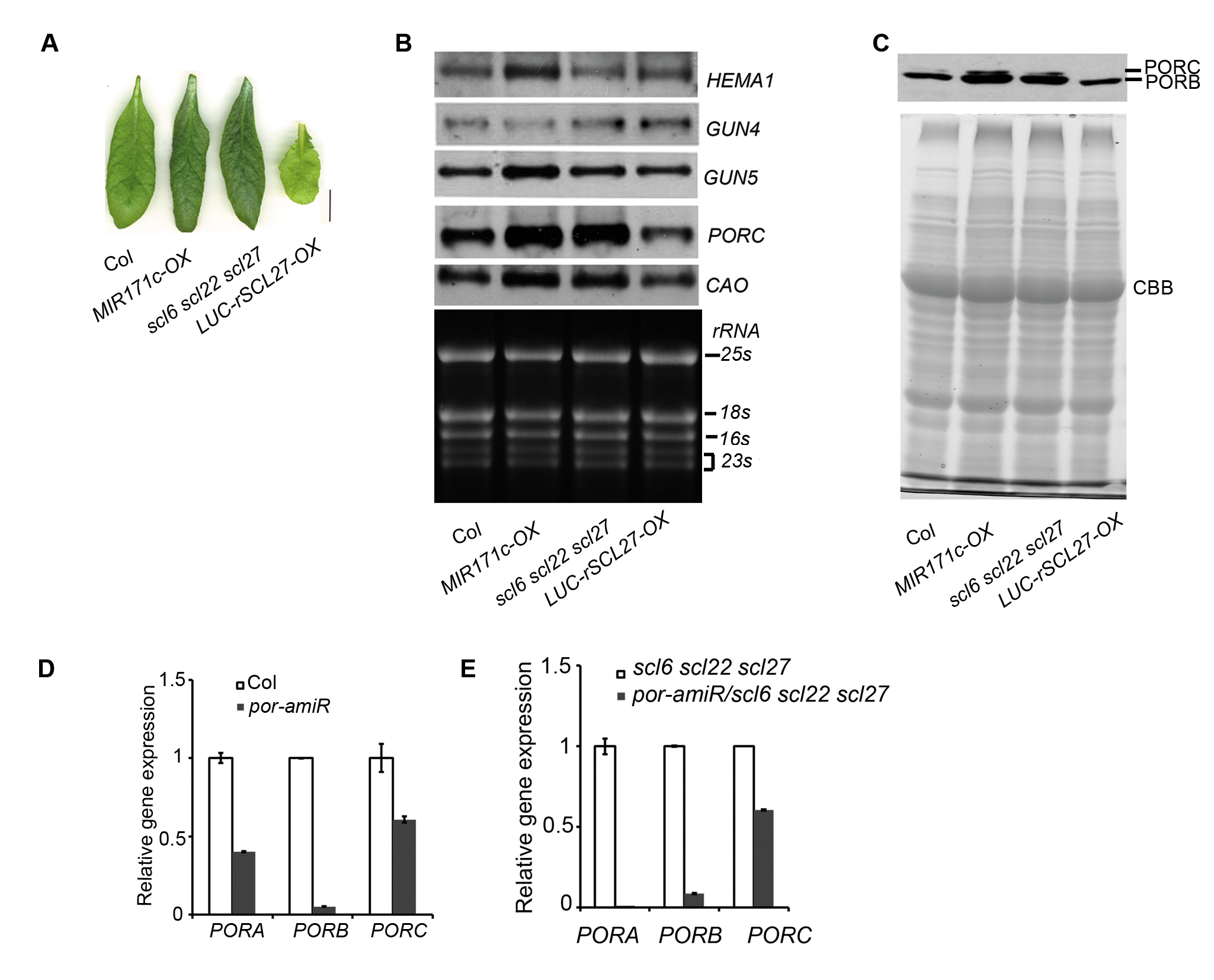

Supplement: Figure S1 — Effect of miR171-targeted SCLs on chlorophyll biosynthesis in light. (A) Leaf phenotypes of WT, MIR171c-OX, scl6 scl22 scl27 triple mutant and LUC-rSCL27-OX plants grown in long-day conditions. Bar = 0.5 cm. (B) Northern blot analysis of the expression of the indicated genes in (A). Five micrograms of total RNA were loaded on each lane. The levels of rRNAs stained with ethidium bromide are shown as loading controls. (C) Immunodetection of POR levels in (A). (D and E) Relative expression levels of PORA, PORB, and PORC genes in Col and por-amiR (D), in scl6 scl22 scl27 and por-amiR/scl6 scl22 scl27 (E). Expression levels were normalized to that of ACTIN2. The expression levels in Col and scl6 scl22 scl27 were set as 1. Error bars indicate s.d. (n = 3). Two biological replicates were performed with similar results. (TIF) [file pgen.1004519.s001.tif]

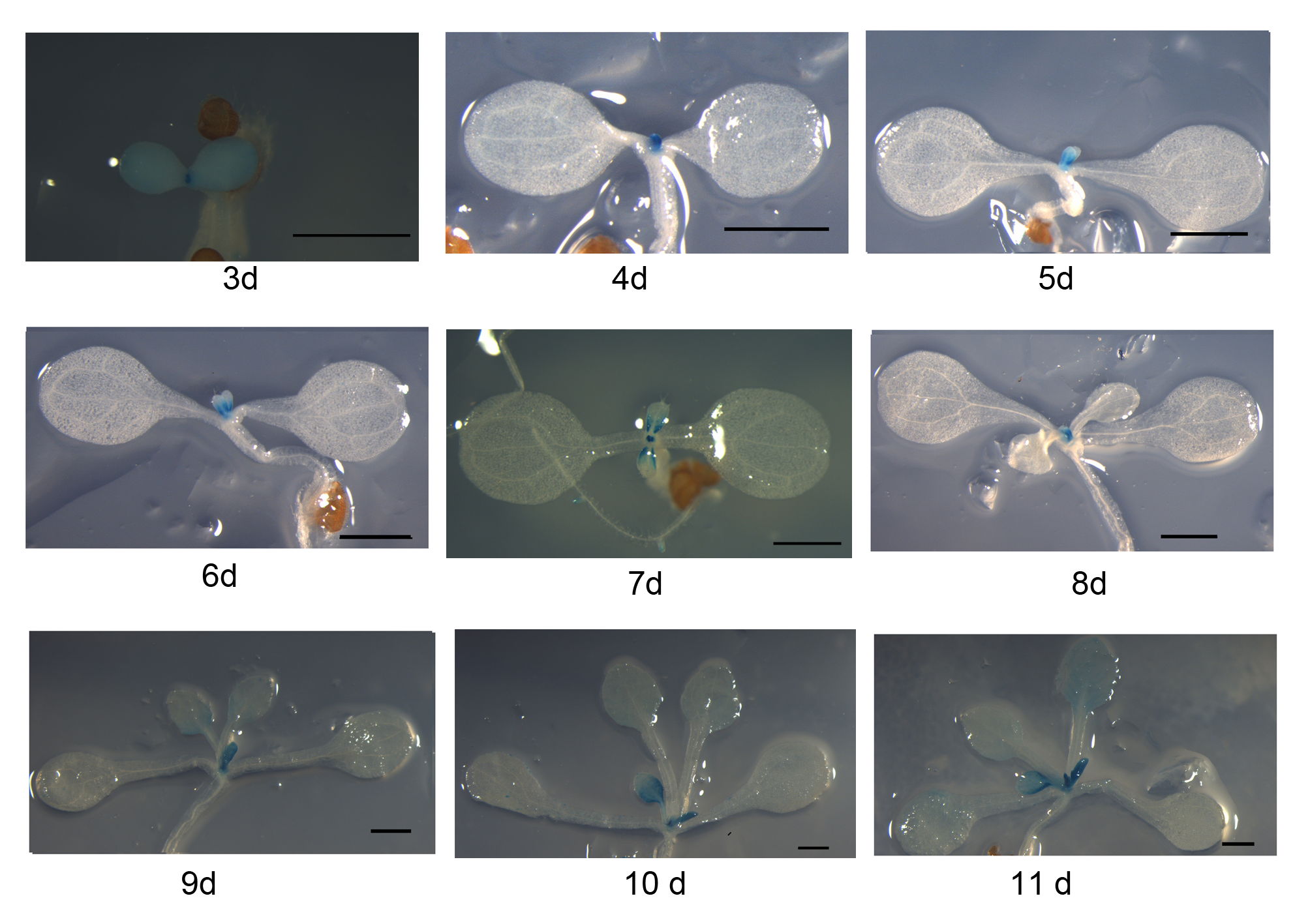

Supplement: Figure S2 — GUS staining of transgenic plants pSCL27::rSCL27-GUS from 3-day to 11-day seedlings. Bars = 1 mm. (TIF) [file pgen.1004519.s002.tif]

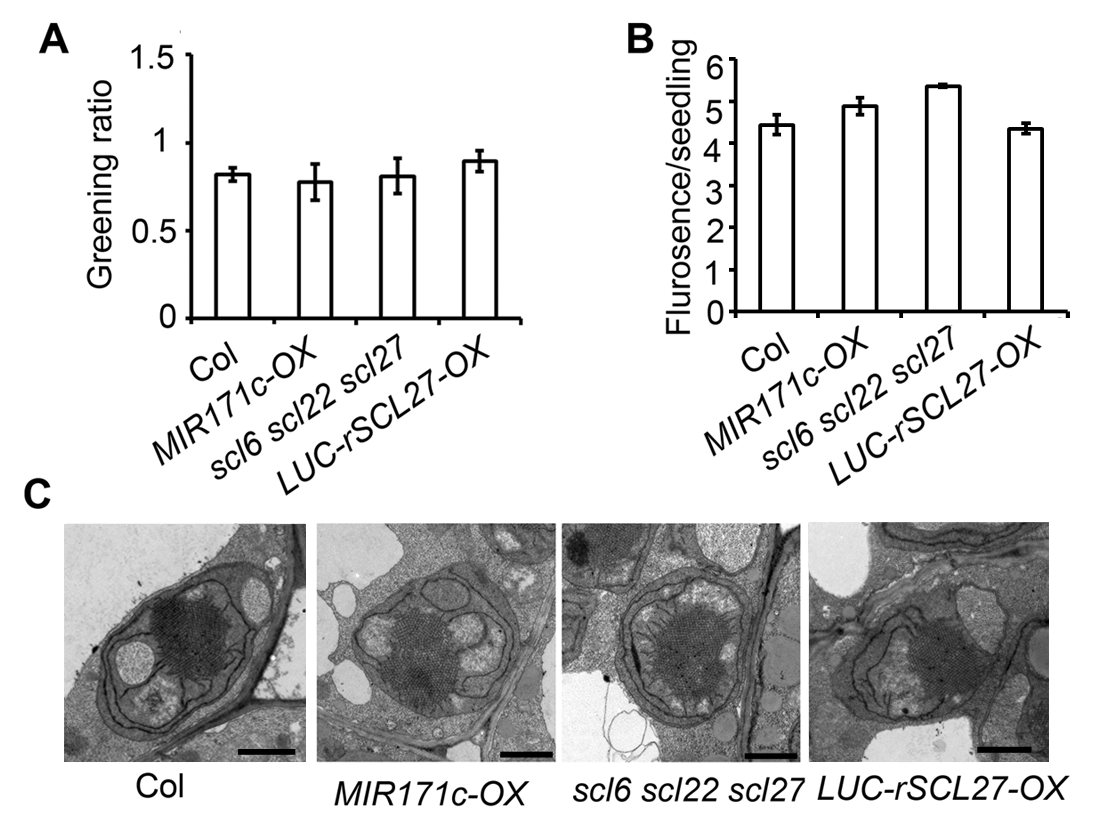

Supplement: Figure S3 — Effect of miR171-targeted SCLs on chlorophyll biosynthesis in the dark. (A) Greening ratio of 5-day-old etiolated seedlings transferred to white light for 2 days. Three biological repeats were performed. Error bars indicate s.d. (n = 30). (B) Pchlide levels of 5-day-old Col, MIR171c-OX, scl6 scl22 scl27, LUC-rSCL27-OX etiolated seedlings. Error bars indicate s.d. (n = 30). Three biological repeats were performed. (C) Ultrastructure of plastids in 5-day-old etiolated seedlings. Bars = 1 µm. (TIF) [file pgen.1004519.s003.tif]

**A**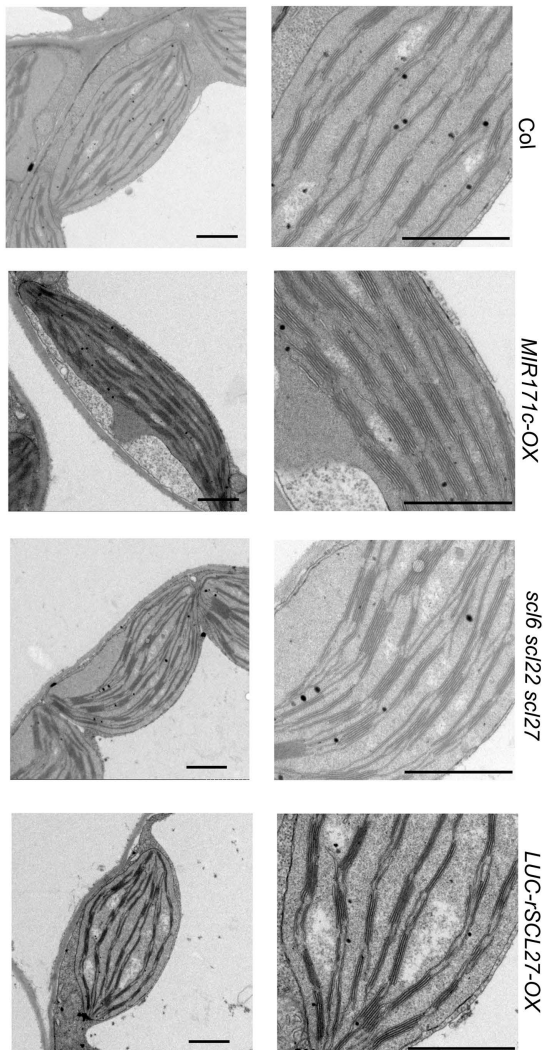**B**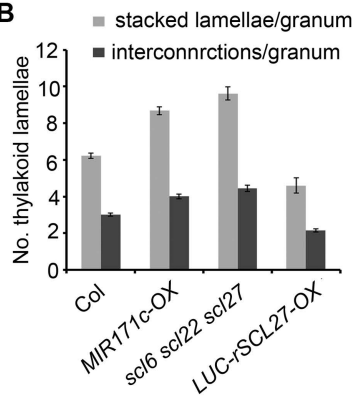**C**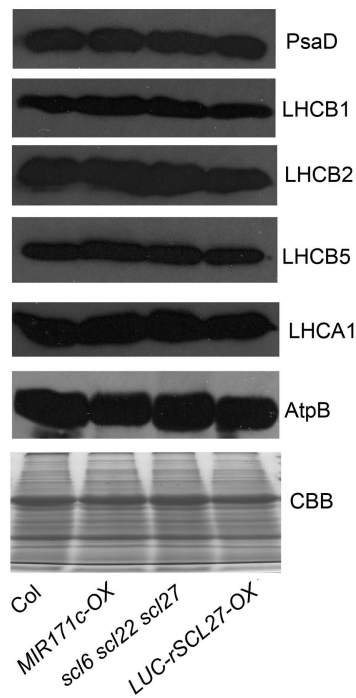

Supplement: Figure S4 — Effect of miR171-targeted SCLs on chloroplast development. (A) Ultrastructure of chloroplasts in mature leaves from 25-day-old plants. Bars = 1 µm. (B) Statistic analysis of stacked and stromal thylakoid membranes. Error bars indicate s.e. (n>110). (C) Immunoblot analysis of PsaD, LHCB1, LHCB2, LHCB5, LHCA1, and AtpB expression in 25-day-old plants. (PDF) [file pgen.1004519.s004.pdf]

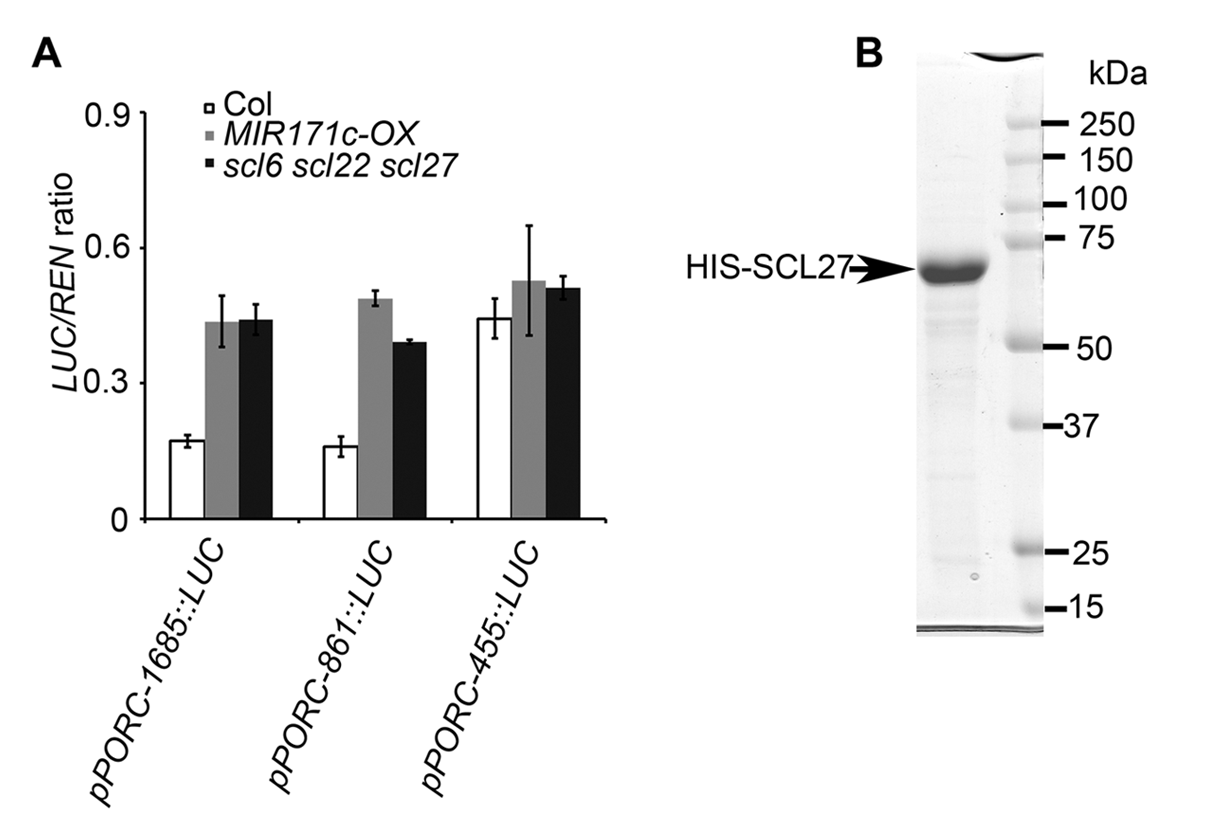

Supplement: Figure S5 — SCL27 binds to the PORC promoter in Arabidopsis. (A) The LUC reporter gene driven by pPORC-1685, pPORC-861 or pPORC-455 was transformed into Col, MIR171c-OX, and scl6 scl22 scl27 plants. The relative LUC activities were normalized to the 35S::REN internal control. Error bars indicate the s.d. (n = 4). Three biological replicates showed similar results. (B) The purified His-SCL27 protein used for EMSA in Figure 3D, 3F, 3G and 4L. (TIF) [file pgen.1004519.s005.tif]

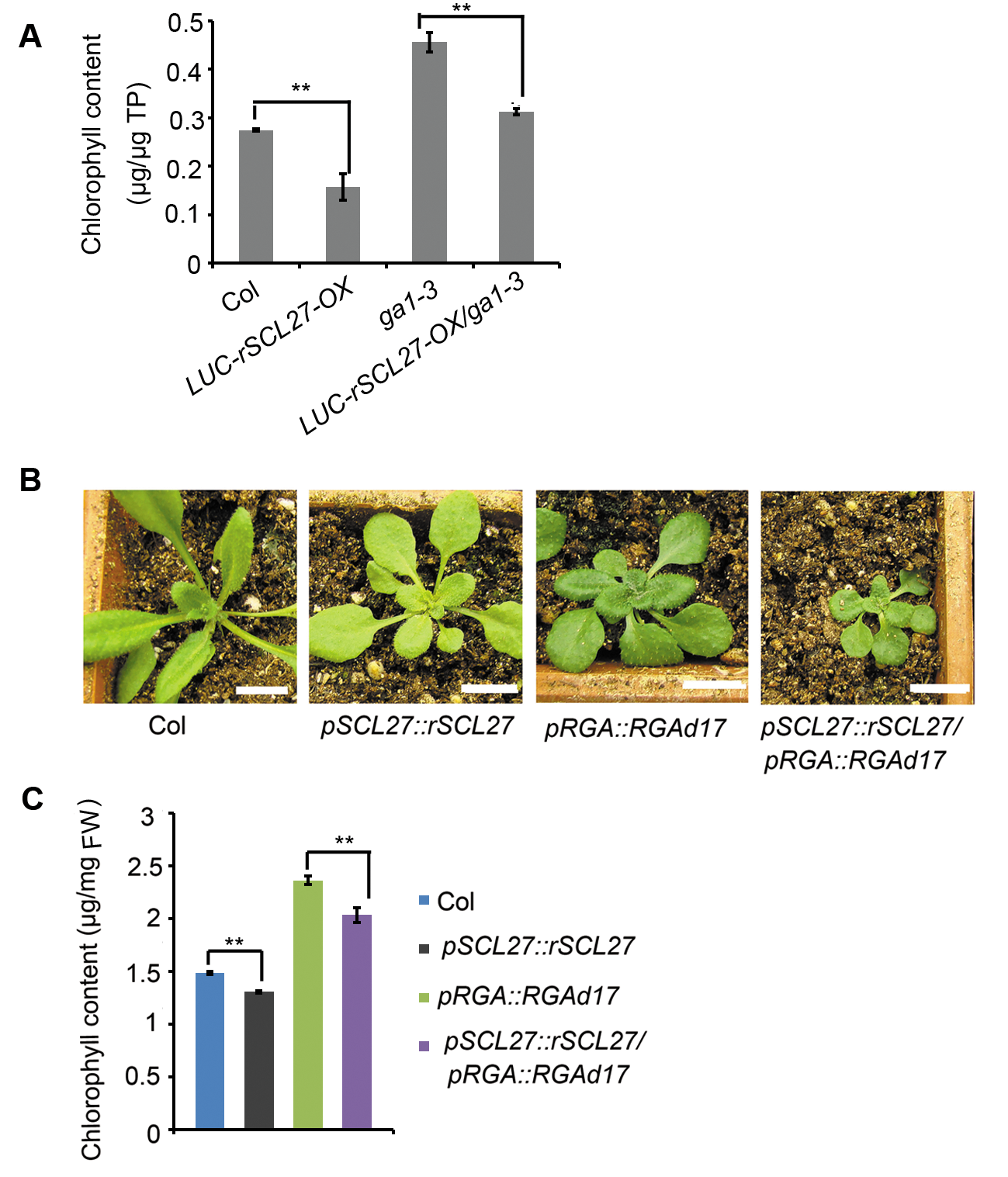

Supplement: Figure S6 — Genetic analysis of SCL27 and RGA. (A) Chlorophyll content of the genotypes shown in Figure 4A based on the total protein (TP). **represent p values (Student's t-test) <0.01 relative to wild-type and ga1-3, respectively. Error bars indicate s.d. (n = 4). (B) Phenotypes of Col, pSCL27::rSCL27, pRGA::RGAd17, pSCL27::rSCL27/pRGA::RGAd17 plants grown in long-day conditions for 25 days. Bars = 1 cm. (C) Chlorophyll content of the genotypes shown in (B) based on the fresh weight (FW). ** represent p values (Student's t-test) <0.01 relative to wild-type and pRGA::RGAd17, respectively. Error bars indicate s.d. (n = 4). (TIF) [file pgen.1004519.s006.tif]

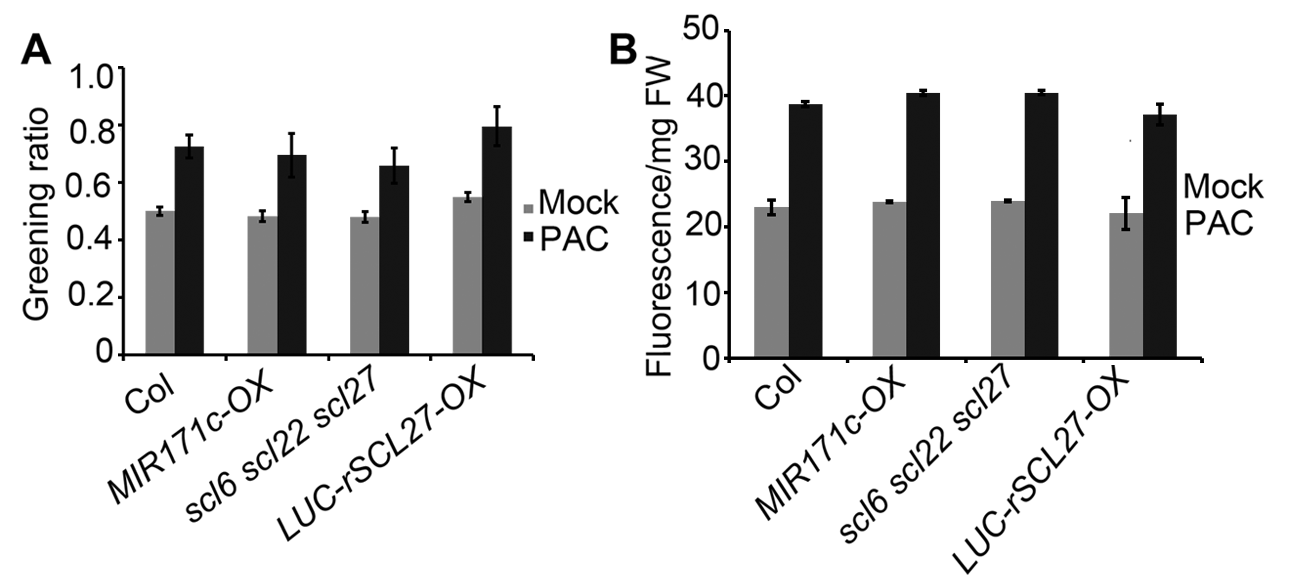

Supplement: Figure S7 — Effect of SCL in DELLA-regulated chlorophyll biosynthesis in the dark. (A) Greening ratio of 5-day-old Col, MIR171c-OX, scl6 scl22 scl27, LUC-rSCL27-OX etiolated seedlings that were grown on the media with PAC or without PAC (methanol, Mock) and transferred to white light for 2 days. Three biological repeats were performed. Error bars indicate s.d. (n = 30). (B) Pchlide levels of 5-day-old Col, MIR171c-OX, scl6 scl22 scl27, LUC-rSCL27-OX etiolated seedlings grown in the media with PAC or without PAC (Mock). Error bars indicate s.d. (n = 3). Three biological repeats were performed. (TIF) [file pgen.1004519.s007.tif]

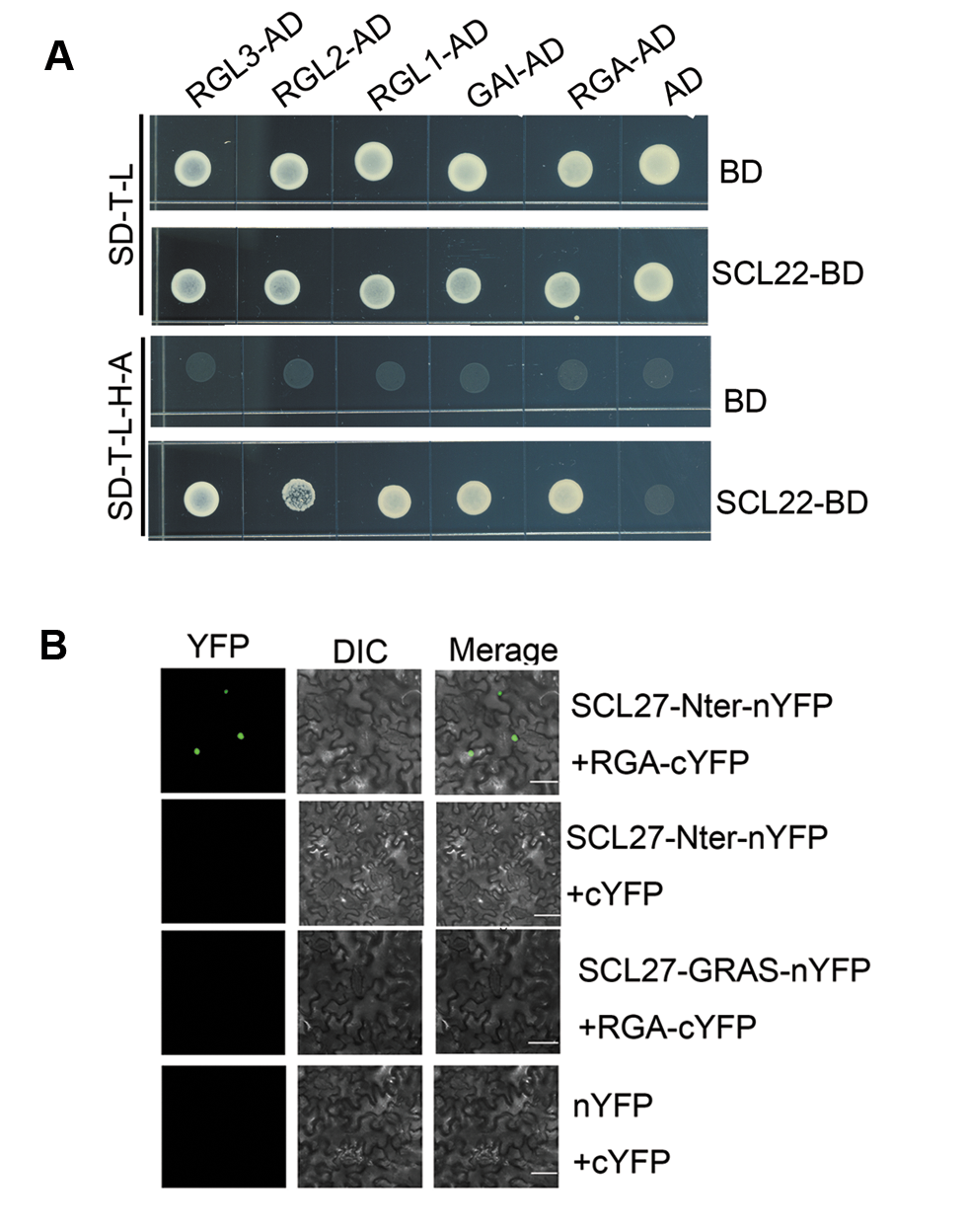

Supplement: Figure S8 — SCLs interact with DELLAs. (A) SCL22 interacts with DELLAs in yeast. (B) BiFC analysis of the interaction between the N-terminal of SCL27 (SCL27-Nter) and RGA. The following pairs of constructs, SCL27-Nter-nYFP and RGA-cYFP, SCL27-Nter-nYFP and cYFP, SCL27-GRAS-nYFP and RGA-cYFP, and nYFP and cYFP, were co-transformed into N. benthamiana leaves. Bars = 50 µm. (TIF) [file pgen.1004519.s008.tif]

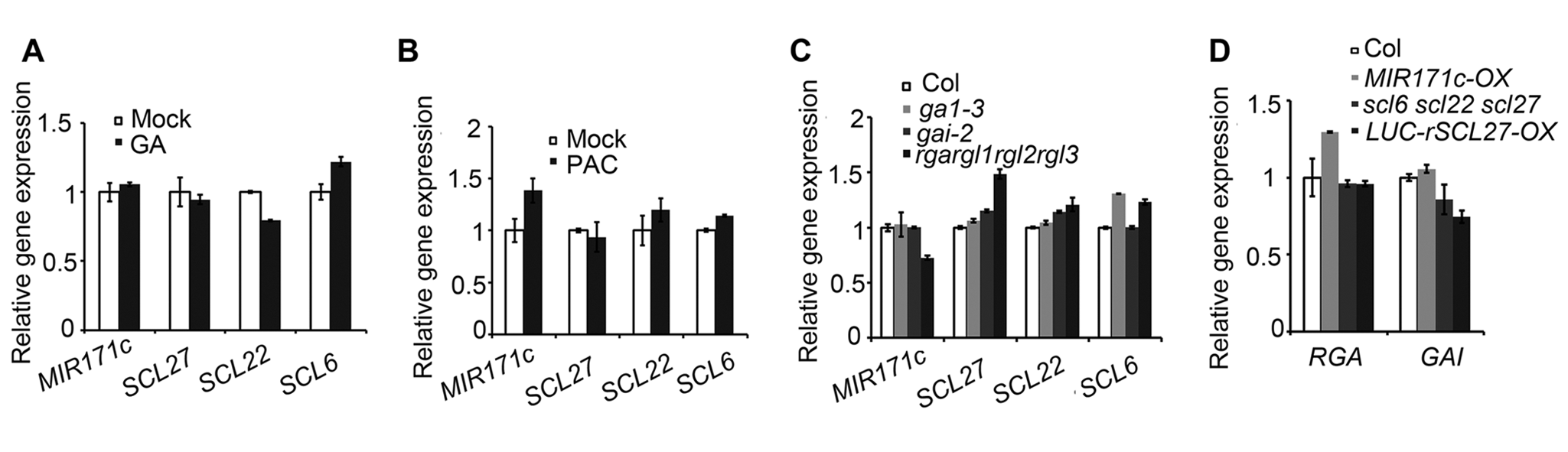

Supplement: Figure S9 — Expression of SCLs and DELLAs is not affected mutually at the transcriptional level. (A and B) qPCR analysis of MIR171c, SCL27, SCL22, and SCL6 expression in seedlings treated with GA3 or Mock (ethanol) (A), and PAC or Mock (methanol) (B). (C) qPCR analysis of MIR171c, SCL27, SCL22, and SCL6 expression in GA mutants including ga1-3, gai-2 and rga rgl1 rgl2 rgl3. (D) qPCR analysis of RGA and GAI expression in Col, MIR171c-OX, scl6 scl22 scl27, LUC-rSCL27-OX seedlings. Expression was normalized to that of ACTIN2 and in WT treated with mock or in WT was set as 1 for each gene. Two biological replicates were performed with similar results. Error bars represent s.d. (n = 3). (TIF) [file pgen.1004519.s009.tif]

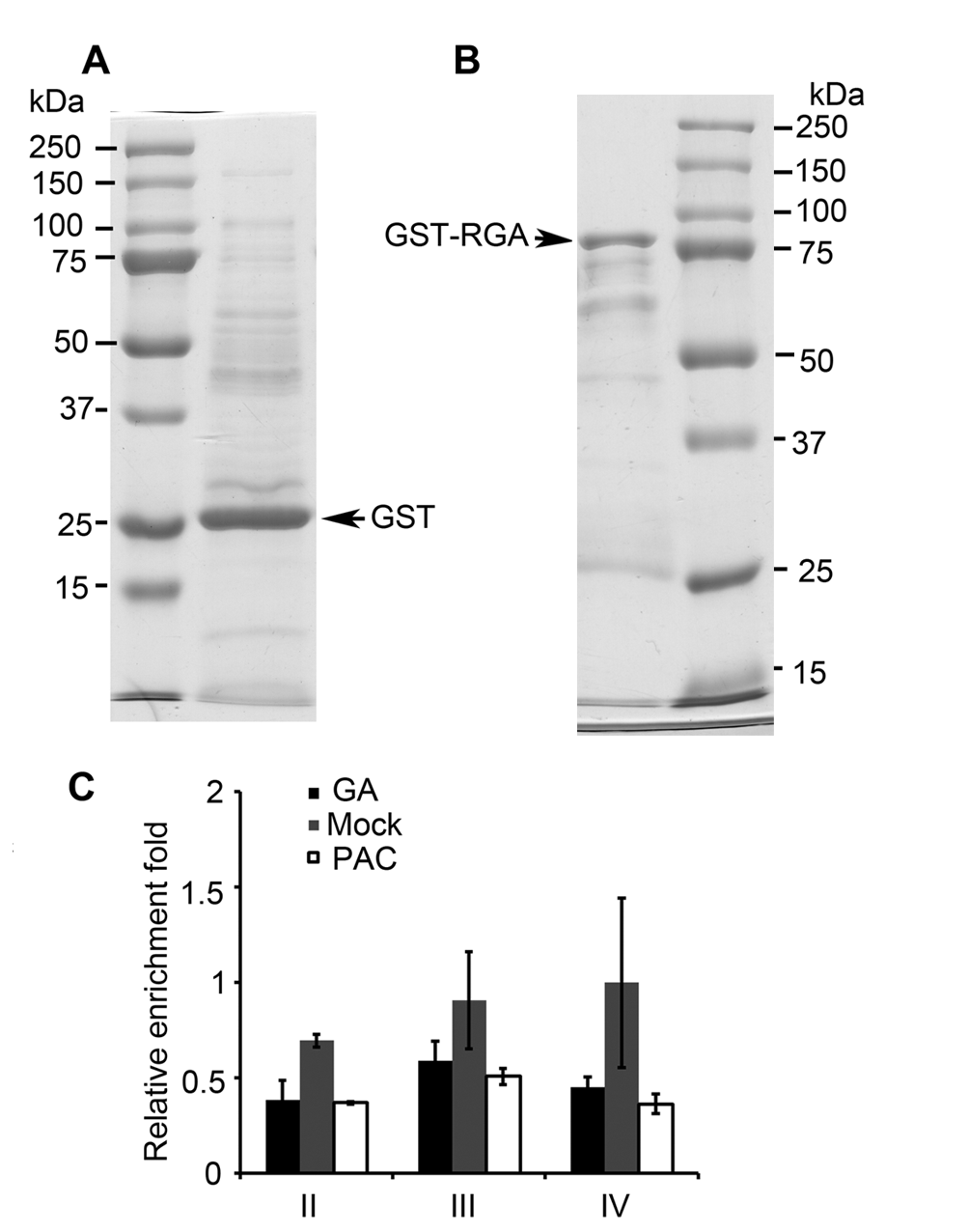

Supplement: Figure S10 — RGA reduces the binding activity of SCL27 to the PORC promoter. (A and B) The purified GST and GST-RGA proteins used for EMSA in Figure 4L. (C) Relative enrichment of the DNA fragments in the immuno-precipitate related to Figure 4M. Leaves of three-week-old Col plants treated with GA, PAC and Mock (without GA and PAC) were used for ChIP experiments. The obtained DNA fragments were quantified via qPCR. The β-TUBULIN-2 promoter was used as a reference. Error bars indicate the s.d. (n = 3). Two biological replicates were performed with similar results. (TIF) [file pgen.1004519.s010.tif]
