# Supplementary material for: Arabidopsis miR171-Targeted Scarecrow-Like Proteins Bind to GT cis-Elements and Mediate Gibberellin-Regulated Chlorophyll Biosynthesis under Light Conditions
Source: PLoS Genet. 2014 Aug 7;10(8):e1004519. doi: 10.1371/journal.pgen.1004519 (PMC4125095; doi:10.1371/journal.pgen.1004519)
Supplement: Table S1 — A list of primers used in this study. (DOCX) [file pgen.1004519.s011.docx]

**Table S1 A list of primers used in this study.**

| Primer | Sequence 5’ to 3’ | Purpose |
| --- | --- | --- |
| POR I miR-s | gaTGGTGGTACATTACCTGCCTAtctctcttttgtattcc | *por-amiR* |
| POR II miR-a | gaTAGGCAGGTAATGTACCACCAtcaaagagaatcaatga | *por-amiR* |
| POR III miR*s | gaTAAGCAGGTAATGAACCACCTtcacaggtcgtgatatg | *por-amiR* |
| POR IV miR*a | gaAGGTGGTTCATTACCTGCTTAtctacatatatattcct | *por-amiR* |
| AmiA | CACCCTGCAAGGCGATTAAGTTGGGTAAC | *por-amiR* |
| AmiB | GCGGATAACAATTTCACACAGGAAACAG | *por-amiR* |
| MIR171ApGreen-F | CCGCTCGAGgtattgcattcataatacagaggtgc | Dual-LUC |
| MIR171ApGreen-R | CGGGATCCAAAGGGACTCTCTCATGCTTAAAGTG | Dual-LUC |
| PORCproPgreen-F-1 | CCCTCGAGGGCCTCCTCTACCTGCAAACGTTTTC | Dual-LUC |
| PORCproPgreen-F-2 | CCCTCGAGGGGAGCTAAAGTTCTAATTCGATTTC | Dual-LUC |
| PORCproPgreen-F-3 | CCCTCGAGGGGAGGCAAAAATGGAAATTTTCATTG | Dual-LUC |
| PORCproPgreen-R | CGGGATCCCGTGTTGTACGGAACTGAAGGTGC | Dual-LUC |
| SCL27 topo-F | CACCATGCCCTTATCCTTTGAAAGGTTTC | Yeast-two-Hybrid |
| SCL27 topo-R | CTAACATTTCCAAGCAGAGACAGTAAC | Yeast-two-Hybrid |
| SCL27-Nter-R | CTAGACTGACTGGTCATCGCCGGC | Yeast-two-Hybrid |
| SCL27-GRAS-F | CACCATGATCATCGAGCAGCTGTTCAACG | Yeast-two-Hybrid |
| RGA topoF | CACCATGAAGAGAGATCATCACCAATTCC | Yeast-two-Hybrid |
| RGA topo-R | TCAGTACGCCGCCGTCGAGAG | Yeast-two-Hybrid |
| SCL27-YFP-*EcoR*I-F | CGGAATTCATGCCCTTATCCTTTGAAAGGTTTC | BiFC |
| SCL27-Nter-YFP-*Sal*I-R | GCGTCGACGACTGACTGGTCATCGCCGGCCATT | BiFC |
| SCL27-GRAS-YFP-*EcoR*I-F | CGGAATTCATGATCATCGAGCAGCTGTTCAACG | BiFC |
| SCL27-YFP -[*Sal*I](http://cid-b12e91df48154caa.spaces.live.com/products/productR0138.asp)-R | GCGTCGACACATTTCCAAGCAGAGACAGTAACAAG | BiFC |
| RGA-YFP-[*BamH*I](http://cid-b12e91df48154caa.spaces.live.com/products/productR0136.asp)-F | CGGGATCCATGAAGAGAGATCATCACCAATTCCAAG | BiFC |
| RGA-YFP-[*Spe*I](http://cid-b12e91df48154caa.spaces.live.com/products/productR0133.asp)-R | GGACTAGTGTACGCCGCCGTCGAGAGTTTC | BiFC |
| SCL27-HIS-F | CCGAATTCCATGCCCTTATCCTTTGAAAGGTTTC | Protein expression |
| SCL27-HIS-R | CGAGTCGACCTAACATTTCCAAGCAGAGACAGTAAC | Protein expression |
| SCL27-pro-F-*EcoR*I | CGGAATTCCACTAATAAGTCCGAACCCTG | Genomic fragments |
| SCL27-pro-R-*Sac*I | CCCGAGCTCCGCCTCCTCAACAACACAGAGT | Genomic fragments |
| SCL27-F-*Xba*I | GCTCTAGATGCCCTTATCCTTTGAAAG | MYC fused |
| SCL27-R-*Sal*I | ACGCGACCTAACATTTCCAAGACGAGAC | MYC fused |
| SCL22-F-*Xba*I | GCTCTAGAATGCCCCTGCCCTTTGAGCA | MYC fused |
| SCL22-R-*Sal*I | ACGCGTCGACTTAACATTTCCAAGCTGAGACAG | MYC fused |
| MIR171c-F-*Kpn*I | GGGGTACCATTTTGTTTGATATTTATTATTTC | Overexpression |
| MIR171c-R-*BamH*I | CGGGATCCTAATTAAAGTTCAGAATATACAC | Overexpression |
| GAI qPCR-F | CACCTCGGCTTGGAAACTCTC | qPCR |
| GAI qPCR-R | TGACAAAGGGAAAAACAGTAGGATTT | qPCR |
| RGA qPCR-F | AATAGTGGCCAAGGTTATCGTG | qPCR |
| RGA qPCR-R | AGTGTGCCAACCCAACATC | qPCR |
| PORB qPCR-F | CGAGAGCACATTCCTCTCTTC | qPCR |
| PORB qPCR-R | GCTTGGATCACTCACCACCT | qPCR |
| PORC qPCR-F | CACGAGGAAACAGGAGTCACG | qPCR CHIP  region IV |
| PORC qPCR-R | CGGAAAAAGAAGCCGAAACA | qPCR CHIP  region IV |
| SCL27 qPCR-F | GTTAGATTTTATTTTGCTCTTTTTGAG | qPCR |
| SCL27 qPCR-R | CCTAAAAAACATATCTCTTTAACAATTTC | qPCR |
| SCL22 qPCR-F | GAGAGCATTTTTTATGAAGATCATCAC | qPCR |
| SCL22 qPCR-R | CACTCGGTATTAATAACAATCTTTCTTC | qPCR |
| SCL6 qPCR-F | GACAAAGGCAAAATGATAGGAAAC | qPCR |
| SCL6 qPCR-R | GTTGTTGTGATGTGGTGTGG | qPCR |
| GUN4 qPCR -F | TCTGCTTCTTCCACCTCCTC | qPCR |
| GUN4 qPCR -R | GAGGCGTTTGTGGTTGAAA | qPCR |
| HEMA1 qPCR-F | TCAAGAACTCTGCAGCAGCTGATCG | qPCR |
| HEMA1 qPC-R | GCAAGCTTCTCACGCATCTCA | qPCR |
| CHLH qPCR -F | ATGGGGTGAGCTCAAAAGAA | qPCR |
| CHLH qPCR-R | TCAGGTGGGAAACTGAAAACA | qPCR |
| CAO qPCR-F | CTAAAACCCGATCCTCTTTTCTCTC | qPCR |
| CAO qPCR-R | CTCTTCTTCTCAACTAATCCACTCTC | qPCR |
| MIR171A qPCR-F | GATATTGGCCTGGTTCACTC | qPCR |
| MIR171A qPCR-R | CCACAAAGTCCAAAATAGAG | qPCR |
| MIR171B qPCR-F | GGAGCTAAGTGGAGATTATAG | qPCR |
| MIR171B qPCR-R | GGTTATAATAACTATCTTTGCC | qPCR |
| MIR171C qPCR-F | TGAGCGCACTATCGGACATC | qPCR |
| MIR171C qPCR-R | TAAACGCGTGATATTGGCAC | qPCR |
| GUN4 Northern- F | CCCTCAAACAACCCACTTCC | Northern |
| GUN4 Northern-R | GGCGTTTCATCGTTAAGCTCC | Northern |
| HEMA1 Northe-F | GGATCTTGTGTTGGTGAAGTAGAAAC | Northern |
| HEMA1 Northen-R | CCTCGGCTTAAGTCATCAACC | Northern |
| GUN5 Northern-F | ATGGCTTCGCTTGTGTATTCTCC | Northern |
| GUN5 Northern-R | CCTTAACTTTAATCGCCAATTCC | Northern |
| CAO Northern-F | GTTCATATATCTGAAGCAAGGGTTTC | Northern |
| CAO Northern-R | CATGGTACGGACATTGAATACGTC | Northern |
| PORB Northern-F | CCTCCATTACCGACCAAATCAAATC | Northern |
| PORB Northern-R | GAAATAAACCGCAGCATTGCAGAC | Northern |
| PORC Northern-F | CTTCCTTCTACCATTTCAATCCAAAAAG | Northern |
| PORC Northern-R | GTTCTGTTCTCCGGAAATTTTCAACG | Northern |
| PORC ChIP -1F | TGGGCTCAAAAAGGTTTCTCTAAAAG | ChIP region II |
| PORC ChIP -1R | ATCAATCATAACGATTTTTGCTTGTG | ChIP region II |
| PORC ChIP -2F | CCTGTTATAGGCATATGAACAAGAC | ChIP region III |
| PORC ChIP -2R | CACTTTTGGATCACACAGCTCTCTTC | ChIP region III |
| PORC ChIP -3F | GATTAAAGAAACGGGTGGAATACAC | ChIP region I |
| PORC ChIP -3R | CATCTCATGAAGTTCAGGAAGCGTAG | ChIP  region I |
| EMSA1-F | AGCCAGTGGCGATAAGCCTTAGTCAAATTTGG | EMSA region II |
| EMSA1-R | AGCCAGTGGCGATAAGATCAATCATAACGATTTTTGCTTGTG | EMSA region II |
| EMSA2-F | AGCCAGTGGCGATAAGCCTGTTATAGGCATATGAACAAGAC | EMSA region III |
| EMSA2-R | AGCCAGTGGCGATAAGCACTTTTGGATCACACAGCTCTCTTC | EMSA region III |
| EMSA3-F | AGCCAGTGGCGATAAGGATTAAAGAAACGGGTGGAATACAC | EMSA region Ι |
| EMSA3-R | AGCCAGTGGCGATAAGCATCTCATGAAGTTCAGGAAGCGTAG | EMSA region Ι |
| b-TUB2-F | TAAATTCTGAACCCATTGTTTCA | ChIP control |
| b-TUB2-R | AGTCCGATGATTGGCTTTATTAC | ChIP control |
